# The global and regional air quality impacts of dietary change

**DOI:** 10.1038/s41467-023-41789-3

**Published:** 2023-10-06

**Authors:** Marco Springmann, Rita Van Dingenen, Toon Vandyck, Catharina Latka, Peter Witzke, Adrian Leip

**Affiliations:** 1 Environmental Change Institute, University of Oxford, Oxford, UK; 2 Centre on Climate Change and Planetary Health, London School of Hygiene and Tropical Medicine, London, UK; 3 European Commission, Joint Research Centre (JRC), Ispra, Italy; 4 European Commission, Joint Research Centre (JRC), Seville, Spain; 5 Department of Economics, KU Leuven, Leuven, Belgium; 6 Institute for Food and Resource Economics, University of Bonn, Bonn, Germany; 7 EuroCARE-Bonn, Bonn, Germany; 8 European Commission, DG Research & Innovation, Bioeconomy and Food Systems Unit, Brussels, Belgium

**Keywords:** Sustainability, Agriculture, Atmospheric chemistry, Risk factors, Socioeconomic scenarios

## Abstract

Air pollution increases cardiovascular and respiratory-disease risk, and reduces cognitive and physical performance. Food production, especially of animal products, is a major source of methane and ammonia emissions which contribute to air pollution through the formation of particulate matter and ground-level ozone. Here we show that dietary changes towards more plant-based flexitarian, vegetarian, and vegan diets could lead to meaningful reductions in air pollution with health and economic benefits. Using systems models, we estimated reductions in premature mortality of 108,000-236,000 (3-6%) globally, including 20,000-44,000 (9-21%) in Europe, 14,000-21,000 (12-18%) in North America, and 49,000-121,000 (4-10%) in Eastern Asia. We also estimated greater productivity, increasing economic output by USD 0.6-1.3 trillion (0.5-1.1%). Our findings suggest that incentivising dietary changes towards more plant-based diets could be a valuable mitigation strategy for reducing ambient air pollution and the associated health and economic impacts, especially in regions with intensive agriculture and high population density.

## Abstract (web summary)

Food production, especially of animal products, is a major source of air pollutants. Here, the authors quantify the impacts dietary changes towards more plant-based diets could have for air quality, labour productivity, and human health.

## Introduction

The food system is a major cause of ambient air pollution, with significant impacts on human health^1–3^. Of particular importance are ammonia emissions that are generated when manure and other fertilizers are handled and applied to fields^4^. Through the formation of ammonium salts, ammonia contributes to the concentration of air-borne fine particular matter, including particles with a diameter smaller than 2.5 micrometres (PM2.5). Such particles are linked to a range of health impacts, including cardiovascular and respiratory diseases^1,5–7^. Another relevant source of agricultural air pollution is methane that is produced, among others, by enteric fermentation (digestion) in ruminant animals and, to a lesser extent, under anaerobic conditions in rice paddy fields^8^. Methane contributes to the formation of ground-level ozone which affects the human respiratory system under prolonged exposure^1,7^. In addition to clinical health impacts, air pollution has been linked to reduced cognitive and physical performance, which has implications for employment opportunities and labour productivity^9^.

Controlling agricultural emissions can make a substantial contribution to reducing the health and economic burden of air pollution^2,3^. Because the majority of food-related air pollutants are associated with the production of animal products^10,11^, a particularly important mitigation option could be dietary changes towards more plant-based diets. Diets containing lower amounts of animal products have been associated with a range of environmental and health benefits. For instance, lower fertilizer requirements can contribute to reduced water pollution, less methane and nitrous oxide emissions can help mitigating climate change, and healthy diets imply reduced mortality from diet-related diseases^11–13^. More recently, regional studies focused on the US, the EU, and China^14–17^ have revealed the diet-related mitigation potential of ambient air pollution, but more work is needed from a global perspective. Existing studies have analysed the impacts of removing different proportions of agricultural emissions from their assessments, but without linking those to concrete dietary interventions or implied behavioural changes^2,3^.

Here we quantify the global and regional impacts of dietary changes on air quality and the related health impacts from air pollution. The dietary changes we consider are towards a set of predominantly plant-based dietary patterns that have been shown to be healthier and more sustainable than current ones (e.g., by being associated with improvements in nutritional adequacy, reductions in dietary risks and mortality, and lower environmental resource use and pollution with respect to greenhouse gas emissions, land use, and water pollution)^11–13^ and are in line with science-based recommendations that combine both human and planetary health^18^. The dietary patterns include nutritionally balanced flexitarian diets with low levels of animal-source foods, as well as vegetarian and vegan diets in which either meat or all animal-source foods were replaced by a mix of legumes and fruits and vegetables. We consider these scenarios as illustrative “what if” exercises intended to provide inputs into decision-making. Our focus is on the relative changes in emissions, air pollution, and health impacts that are associated with dietary changes towards this set of dietary patterns.

We followed a multi-model approach to analyse the air pollution impacts of dietary changes (see the “Methods” section). First, we used a global agriculture-economic model to estimate what impacts dietary changes would have on agricultural production and the associated emissions of precursors to air pollutants, including ammonia and methane. Second, we used an air-quality model specifically calibrated for scenario-based analyses to estimate what impacts changes in ammonia and methane would have on the concentration of PM2.5 and ground-level ozone. Third, we used epidemiological exposure–response relationships to estimate the health impacts of changes in air pollution. Fourth, we used economic models to quantify the monetary value of the health benefits and the potential impacts on labour productivity. For the analysis, we assumed the dietary changes to occur over the current decade and show the associated impacts in the year 2030 as compared to a business-as-usual development pathway for income and population levels. We include additional analyses for the years 2010 and 2050 in the Supplementary Information. The global coverage of our assessment accounts for the geographical production and trade patterns of food products and captures the transboundary effects of air pollution and the associated economic consequences.

## Results and discussion

### Agricultural emissions

**Fig. 1:**
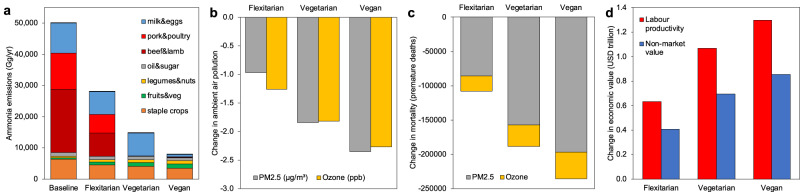
Global air quality impacts of dietary change. The impacts include global changes in agricultural emissions (**a**), air pollution (**b**), premature mortality (**c**), and economic output (**d**) in the year 2030 for dietary changes to flexitarian, vegetarian, and vegan diets. Uncertainty intervals for the health and economic estimates are listed in the SI Datafile. PM2.5 denotes particular matter with a diameter smaller than 2.5 micrometres. The concentration of PM2.5 is measured in micrograms per cubic metre (µg/m³) and that of ozone in parts per billion (ppb).

In our analysis, dietary changes towards more plant-based diets had a substantial impact on food intake and production (SI Tables 12, 13, SI Figs. 3 and 4), as well as on agricultural emissions and air pollution. In our baseline projections for 2030, livestock production was responsible for the majority (80–84%) of all food-related ammonia and methane emissions (Fig. 1a, SI Table 14), with animal source foods having 10 to up to 1000 times the emissions footprints of plant-based foods (SI Table 2). Dietary changes towards lower consumption of animal source foods therefore substantially reduced agricultural emissions—by 84–86% globally for the adoption of vegan diets, 69–70% for vegetarian diets, and 44–48% for flexitarian diets.

Across regions, the reductions in agricultural emissions were particularly large where livestock production is responsible for a large share of emissions (SI Fig. 5). These regions include Latin America (e.g. 61–90% across the diet scenarios for ammonia), Developed Asia-Pacific countries including Australia and New Zealand (59–86%), as well as North America (53–73%) and Europe (53–86%). Regions with relatively low meat-related emissions, such as Africa, Southern Asia, and South-East Asia, had less saving potential and exhibited less substantial reductions in emissions (22–89%, 24–80%, and 31–86%, respectively).

### Air pollution

The changes in agricultural emissions affect air pollution through atmospheric reactions and transportation. The globally averaged exposure to PM2.5 was reduced by 3% for flexitarian diets, 6% for vegetarian diets, and 7% for vegan diets, which correspond to reductions in the anthropogenic fraction of 5%, 7%, and 8%, respectively. The exposure to ozone was reduced by 2%, 3%, and 4% for the same set of diets (Fig. 1b, SI Tables 15, 16).

**Fig. 2:**
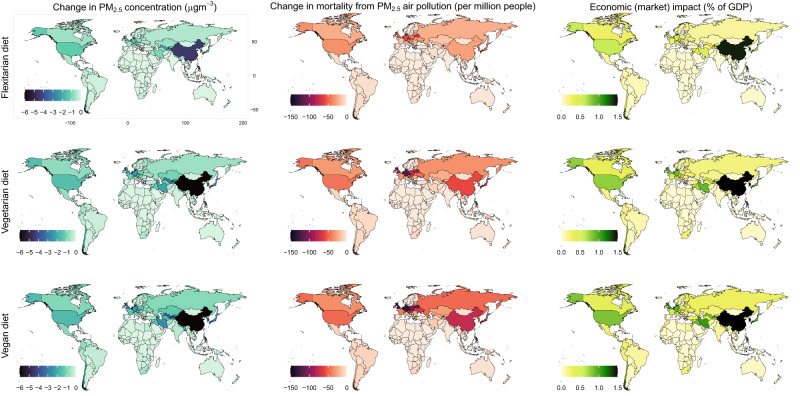
Regional air quality impacts of dietary change. The impacts include changes in particulate matter concentration (*left panels*), mortality from air pollution (*middle panels*), and labour productivity (*right panels*) for the adoption of flexitarian (*top panels*), vegetarian (*middle panels*), and vegan (*bottom panels*) diets. Please note that the economic impacts are truncated at 1.5% of GDP. The maps were produced using the “maps” package in R (https://cran.r-project.org/web/packages/maps/index.html) and Natural Earth data for geographical outlines and borders (https://www.naturalearthdata.com/about/terms-of-use/).

Across regions, reductions in PM2.5 were largest in regions with large reductions in ammonia emissions and where ammonia emissions are the main contributor to PM2.5 due to emissions control measures in other sectors (Fig. 2, SI Figs. 6 and 7, SI Table 15). Those regions included North America (14–16% across the diet scenarios; 1.2–1.6 µg/m³), Developed Asia-Pacific (12–21%; 1.3–2.1 µg/m³), and Eastern Asia (11–25%; 4.3–10.3 µg/m³). Reductions in PM2.5 were lowest in Africa (0.0-0.1 µg/m³, 0%) and in Southern and South-East Asia (0.0–0.4 µg/m³; 0-1%).

The regional differences in ozone exposure were less pronounced because its precursor, methane, has a longer lifetime and therefore equilibrates more globally in concentration. Regional formation of surface-level ozone is therefore more dependent on oxidant availability and photochemical processes^19,20^, with greater responses in the Middle East (a reduction of 2.1–3.8 ppb, 3-5%), Europe (1.7–3.0 ppb, 3–6%) and North America (1.5–2.7 ppb, 3–5%), and the lowest in parts of Asia (1.1–2.1 ppb, 1–4%) (SI Table 16).

### Health and economic impacts

The diet-related improvements in air quality were associated with reductions in premature mortality. In line with the changes in air quality, adoption of vegan diets was associated with the greatest global reduction in premature mortality (236,000 avoided deaths, 95% confidence interval (CI) 176,000–324,000; corresponding to a 6% reduction in all premature deaths attributable to PM2.5 and ozone), followed by vegetarian diets (188,000 avoided deaths, CI 141,000–257,000; 5%), and flexitarian diets (108,000 avoided deaths, CI 79,000–147,000; 3%) (Fig. 1c). Globally, most of the benefit (80–83% across the diet scenarios) was attributable to reductions in PM2.5 related to lower levels of ammonia (SI Table 17).

The magnitude of health benefits differed substantially across regions (Fig. 2, SI Fig 6, SI Table 17). The European region, where intensive agriculture is combined with high population density, exhibited the greatest relative reductions (9–21% fewer premature deaths from PM2.5 and ozone across the diet scenarios, 19,700–44,100), followed by North America (12–18%, 14,400–21,000), and Developed Asia-Pacific (10–18%, 5300–9500), whilst Eastern Asia exhibited large absolute reductions (4–10%, 49,200–120,900).

The diet-related improvements in air quality were associated with enhanced labour productivity, which impacts economic output. Enhanced productivity from clean air increased economic output by about USD 1.3 trillion (with a low to high range of USD 0.5–3.0 trillion, corresponding to 1.1% with a range of 0.4–2.5% of global GDP in 2030) for the adoption of vegan diets, and USD 0.6–1.1 trillion (0.5–0.9% of GDP) for the adoption of flexitarian and vegetarian diets, respectively (Fig. 1d). Valuing the economic benefits of cleaner air using ‘non-market’ estimates that place a value on changes in mortality risk resulted in similar trends and orders of magnitude (0.3%, 0.6%, and 0.7% of GDP for flexitarian, vegetarian, and vegan, respectively) (SI Table 18, SI Figs. 8 and 9). Across regions, the economic gains were particularly large for countries with high economic output, including those in Eastern Asia (increase in economic output of 1.3–3.2% of GDP), North America (0.6–0.8%), Developed Asia-Pacific (0.4–0.6%), and Europe (0.3–0.6%) (Fig. 2, SI Fig. 8).

### Contributions to the literature

Reducing the burden of air pollution is a major public and environmental health challenge. The food system is a large source of air pollutants, most of which are linked to the production of animal-source foods. Our analysis quantified how dietary changes to more plant-based diets can contribute to reducing the health and economic burden of air pollution from the food system. We found that diet-related reductions in air pollution could reduce premature mortality by more than 230,000 deaths globally per year in 2030, which represents a reduction of 6% in the number of premature deaths that are due to air pollution. In regions with intensive agriculture and high population density, the benefits ranged up to 21% of premature deaths due to air pollution for Europe, and 18% for North America and developed Asia-Pacific countries such as Australia and New Zealand, whilst Eastern Asia exhibited gains of 4–10% that were large in absolute terms. The economic value of these changes in mortality amounted to 0.3–0.7 of global GDP, and additional improvements in productivity amounted to another 0.5–1.1% of GDP. For comparison, the total value added of the agricultural sector currently represents <1% of GDP in the USA and in Germany.

The health benefits of dietary changes identified in this study are comparable to existing estimates. Previous global studies estimated that a 50% reduction in agricultural emissions could reduce premature mortality from air pollution by about 200,000–250,000 per year globally^2,3^. Our study advances the identification of concrete measures for reducing air pollution and its associated impacts. Instead of removing different percentages of agricultural emissions without specifying how such changes would occur, we considered concrete options for dietary change, which provides actionable information about how to achieve specific improvements in air pollution. For example, we identified the greatest scope for reducing agricultural emissions and the associated impacts in regions that have concentrated livestock industries coupled with high population densities such as Europe, North America, and developed Asia-Pacific countries. In these regions and in general, we found that the reductions in emissions and impacts increase with increasing ambition of dietary change towards more plant-based diets, ranging from flexitarian diets with moderate amounts of animal source foods (108,000 avoided premature deaths) over vegetarian diets that contain dairy but no meat (188,000 avoided premature deaths) to completely plant-based vegan diets (236,000 avoided premature deaths).

We also complement and further advance previous national case studies^14–17^. Our estimates of health benefits from dietary change are lower than those estimated for the US (10,700–13,100 avoided premature deaths from reduced ammonia emissions in 2015^14^ compared to 5000–5900 in 2010 and 12,000–17,100 in 2030 in our study) and China (55,000–74,800 avoided premature deaths in 2012 and 2010, respectively^16,17^, compared to our estimate of 28,200–52,100 in 2010 and 39,000–103,800 in 2030), and higher than those estimated for Europe (10,700 avoided premature deaths in 2050^15^ compared to 22,400 in our study for the same year). These differences are within expectations as each study relied on different air-pollution models, some of which including ours used reduced-form representations, different diet scenarios and pathways, and different assumptions about market adjustments, including no adjustments^17^, proportional adjustments^14,16^, and inclusion of market feedbacks^15^. For example, the estimates for the US and China did not include the mediating effects of market feedback, whereas the estimates for Europe assumed strong reductions in air pollutants in the baseline pathway to 2050 and considered less ambitious flexitarian diets with larger amounts of animal source foods, resulting in half the reduction in ammonia emissions that we identified. We complement the various national case studies by covering a greater set of diet scenarios, including both particulate matter and ground-level ozone, considering market feedback, including both non-market and market-based valuations, and extending the regional coverage.

That market feedback is important to consider for a consistent analysis of dietary-change impacts can be underlined by comparing changes in food consumption with those in food production (see, e.g., SI Table 26 for comparisons for Europe, the USA, and China). Static analyses without market feedback often apply percentage changes in consumption (derived from comparing current diets to diet scenarios) to production statistics. However, proportional changes in consumption generally do not map one-to-one onto changes in production because (i) a large portion of grain production is used as animal feed so that production changes exceed consumption changes when diets become more plant-based; (ii) some foods are imported to a large degree from abroad (e.g., legumes in Europe, the USA, and China) so that production changes stay below consumption changes; and (iii) some countries/regions are specialized (have a competitive advantage) in producing certain foods (e.g., meat and dairy production in Europe) so that exports mitigate part of the reduction in consumption with the result that production changes stay below consumption changes. Considering market and food-chain feedback is important for resolving these interconnections, and not including them can lead to biased production estimates which further affect the validity of the health and environmental impacts associated with food production.

### Limitations

As with any study, our study has several limitations. First, we focused on a set of specific dietary patterns and did not investigate other agricultural mitigation options. The set of diets was chosen to align with the literature on healthy and sustainable diets and cover a range of options for dietary change, but other low-meat diets can in principle have similar impacts^12,13^. Although it is now recognised that even completely plant-based diets can be appropriate for all life stages, nutrient adequacy can be a concern for at-risk groups and some nutrients (e.g., B vitamins in vegans) that might require access to specific foods and/or supplementation^21^. The scale of dietary changes considered here will also likely require dedicated policy support in terms of multi-component approaches (e.g., fiscal incentives paired with the provision of information)^11,22^, as well as social acceptance, neither of which we considered in our study.

Second, we focused on the production side of the food system and did not consider the additional health and environmental impacts associated with changes in food consumption, storage and transportation^12,23^. We also did not include production-side impacts such as the potential crop yield benefits due to ozone reduction^24^. Adding these additional aspects would further increase the magnitude of the health and environmental co-benefits of reductions in air pollution. In addition, mitigation options related to changes in farm-level management, e.g. of manure and feed, can also make important contributions to reducing the burden of air pollution of the food system^3,25^, albeit to a lesser degree than the types of dietary changes considered here and elsewhere^14^.

Third, our multi-model analysis combined state-of-the-art methods of analysis with a broad scope, but we were not able to include all aspects relevant to the economic impacts of air pollution and dietary changes. These include analyses of price and expenditure changes and whether the considered diets can be affordable for vulnerable groups^26^. And although we were able to include more market feedback than most existing studies, we were not able to model a complete alignment of market conditions with the diet scenarios and instead combined the market feedback of an interim scenario with adjustment factors for production, consumption, and agricultural emissions. Such an adjustment approach is in line with existing studies^2,3,14,16,17^, but it did not allow us to analyse the full scope of economic and environmental adjustments, including changes in the composition of fertilisers and explicit analyses of land-use changes.

Fourth, we explicitly resolved the uncertainties regarding the health impacts of changes in air pollution and the economic value of these changes (see the “Methods” section and SI Datafile), but stress that the uncertainties regarding market feedback deserve more attention in future studies, including not only resolving the shifts in food supply chains, but also industry responses. More generally, we would like to note that there are different strategies for investigating the links between food consumption, air pollution, and the related health and economic impacts, ranging from using fully integrated models to linking separate systems models. Systematically quantifying the full uncertainty involved will require a multi-model assessment, as some uncertainty will inevitably relate to model particularities^27^. We think our study makes a contribution to this end, as the scenario description of our analysis can be used to inform the development of standardised model protocols that can be implemented by other global agriculture-economic models and combined with different socio-economic and emissions pathways.

### Implications

Our analysis indicates that diets high in animal-source foods are substantial drivers of air pollution and, compared to healthier and more plant-based diets, could be associated with more than 230,000 premature deaths and economic losses of more than 1% of GDP in 2030. These findings add to the growing literature on the benefits of dietary change towards more plant-based diets. Adoption of more plant-based diets has been estimated to substantially reduce diet-related mortality, as well as food-related greenhouse gas emissions and the demand for agricultural land, water, and fertilizers^10–13,18,28^. Our results highlight that incentivising dietary changes towards healthy and more plant-based diets could also be a valuable mitigation strategy for reducing ambient air pollution and the associated health impacts. As such, policy packages that aim to bring air quality in line with the revised guidelines of the World Health Organisation announced in September 2021 can benefit from a broad perspective that considers demand-side mitigation options. Measures that would support dietary changes towards more plant-based diets include updating national dietary guidelines^13^, providing fiscal incentives that price in the health and environmental costs of foods^29–31^, reforming agricultural subsidies from health and environmental perspectives^32^, and generally integrating health and environmental considerations across domains^33^.

## Methods

We used a multi-model approach that consisted of agriculture, emissions, air quality, health, and economic analyses to estimate the air pollution-related health and economic impacts associated with dietary changes.

### Agricultural analysis

For analysing the impacts of dietary change on agricultural production and emissions, we used the CAPRI agriculture-economic model^34^. The CAPRI model is a partial equilibrium model of the agricultural sector that is extensively used for policy and market assessments in the European Union (EU) and globally^31,35,36^. The model is governed by a system of behavioural equations representing agricultural supply, the demand for food, feed and from processing industries, as well as multilateral trade relations differentiated by commodity and geographical units. It combines a global market model with a regional supply model for agricultural commodities that captures farm and industry-level behaviour with respect to the use of capital and labour, subject to resource constraints, prices, and agricultural policies. Consumer demand is represented by indirect utility functions depending on prices and income. National food consumption in CAPRI is based on food availability data from FAOSTAT and Eurostat^37^. The model solves for a market equilibrium in which global supply matches demand. A more detailed description of the model is provided by Himics and colleagues^35^ and in the Supplementary Information (SI.1).

We used the CAPRI model to estimate the changes in agricultural production and emissions that would be associated with dietary changes towards a set of healthy and sustainable dietary patterns. The dietary patterns were based on those developed by the EAT-Lancet Commission on Healthy Diets from Sustainable Food Systems and included nutritionally balanced flexitarian diets with low levels of animal-source foods, as well as vegetarian and vegan diets in which either meat or all animal-source foods were replaced by a mix of legumes and fruits and vegetables (Supplementary Information, SI.2)^18^. The dietary patterns were regionalised, e.g. by preserving regional preferences for the type of grains and by implementing food-group recommendations as lower and upper values, so that lower than maximum recommended intake, e.g. of red meat, was preserved, as was higher than minimum recommended intake, e.g. of fruits and vegetables^12^.

For representing a time dimension of dietary changes, we projected socio-economic and technological changes to the year 2030 and implemented the dietary changes within that time horizon. Our main projections were based on a middle-of-the-road development pathway (SSP2) and the associated emissions trajectory (RCP 6.0) and climate policies. As our focus is on the relative impacts of dietary change, we did not introduce additional changes in technologies such as changes in production systems that go beyond efficiency improvements over time. SI Table 2 provides an overview of the diet scenarios in 2030, aggregated to the detail with which food groups are represented in CAPRI. In additional analyses (SI Tables 19–22), we estimated impacts for the year 2010, and also considered a longer time horizon to 2050, together with more optimistic socio-economic and emissions trajectories (SSP1, RCP 2.6).

### Emissions analysis

The behavioural equations in agriculture-economic models like CAPRI are best suited to study the impacts of marginal changes in food consumption and agricultural production. To assess the changes in emissions from substantial changes in consumption towards the different diet scenarios, we therefore followed a two-step approach (Supplementary Information, SI.3). First, we shifted CAPRI’s baseline diets to an interim scenario that includes some changes towards the different diet scenarios. Following this “shock” to the baseline, the model adjusts agricultural supply chains, which impacts the relative prices of goods. The change in prices affects demand such that the final demand differs from the initial shocks. For complete alignment with the diet scenarios, we then scaled food demand and the associated emissions in a second step, for which we used the set of region and food-group-specific emissions footprints (emissions per quantity of food demanded) from the first iteration. This way of targeting specific dietary scenarios allowed us to account for market feedback whilst ensuring a valid representation of the different dietary patterns.

We used dedicated emissions modules for quantifying the impacts on air pollution from changes in agricultural demand and production^38,39^. These modules use time series data of national GHG emission inventories, obtained from the Food and Agriculture Organization of the United Nations (FAO), to calibrate an input–output model that generates commodity-specific emission factors. In this way, CAPRI includes emission factors for ammonia (NH_3_) and methane (CH_4_) that are generated from enteric fermentation, manure management, and cultivated soils. Emissions from fodder production are included in the emissions assigned to livestock products, and emissions from marketable feeds (wheat, soy, etc.) are included in the total emissions of crop commodities. This approach makes sure that all emissions are assigned to the region where they occur.

### Air-quality analysis

We used the TM5-FASST air quality model^40^ to estimate the changes in air pollution and associated mortality that result from dietary changes towards healthier and more sustainable diets (Supplementary Information, SI.4). The TM5-Fast Scenario Screening Tool (TM5-FASST) is a global reduced-form air quality source-receptor model that has been designed to compute ambient pollutant concentrations as well as a broad range of pollutant-related impacts on human health and agricultural crop production. It is based on linearized emission-concentration responses derived from the chemistry-transport model TM5^41^. The linearised nature increases computational speed and makes it well suited for comprehensive scenario analysis and is regularly applied in those^24^. To better represent the response sensitivities of changing concentrations of emissions under conditions of strong reductions of NO_*x*_ and NH_3_^42,43^, we included second-order, non-linear correction factors for the formation of ammonium nitrate and sulfate. Model responses have been validated against the full TM5 model^40^.

In TM5-FASST, changes in ammonia emissions affect the share of ammonium nitrate and sulfate in fine particulate matter (PM2.5), and the associated changes in PM2.5 exposure affect human health. In addition, changes in methane concentrations impact background ozone levels, which affects both agricultural production and human health, albeit to a lesser degree^44,45^. For the analysis, we mapped the changes in ammonia and methane emissions from CAPRI to the resolution of TM5-FASST and gap-filled other sectoral emissions by using the emissions data from the Climate Model Intercomparison Project (CMIP6) that matched the set of socio-economic and emissions pathways we used in CAPRI. We then calculated global grid maps of particulate matter and ozone concentration.

### Health analysis

We followed the methodology developed for the 2017 edition of the Global Burden of Disease (GBD) project to evaluate changes in premature mortality associated with changes in ambient air pollution for six causes of death at the level of individual grid cells^5,46,47^. The causes of death linked to particulate matter included chronic obstructive pulmonary disease (COPD), lower respiratory infections (LRI), lung cancer (LC), ischaemic heart disease (IHD), stroke, and diabetes mellitus type 2 (DMT2), and those linked to ozone included COPD. The relative risks for PM2.5 exposure were calculated from the integrated exposure–response functions developed by Burnett and colleagues^5^ with updated functional parameters^47^. The Supplementary Information (SI.4) provides an overview of the relative risk parameter used.

As exposure metrics, we used the annual mean ambient PM2.5 concentration and the seasonal 8 h-daily maximum ozone concentration (i.e., the 6-month period with the highest ozone). To evaluate population exposure at each grid cell, we regridded the native 1° × 1° output resolution to 7.5’ × 7.5’ and overlaid it with population grid maps of the same resolution and for the same target year and socio-economic trajectory as used in CAPRI^48^. This approach captures how trends at the 1° × 1° level affect regions at scales of 7.5’ × 7.5’. To calculate the final impacts on mortality, we used age, cause and country-specific mortality rates^49^, projected to the target year of analysis^50^. We calculated country-level changes in premature mortality attributable to changes in diets by summing over each country’s grid cells.

### Economic analysis

We used two complementary methods to estimate the economic value of the diet-related changes in air pollution and the associated health impacts (Supplementary Information, SI.5). First, we estimated the non-market value of reduced mortality risks, often described as the value of statistical life^28^. We multiplied the avoided premature deaths with the valuation that is based on a comprehensive global meta-analysis of stated preference surveys of mortality risk valuation undertaken for the Organization for Economic Co-operation and Development (OECD)^51^. In line with previous analyses^28^, we used a benefit-transfer method to calculate values of statistical life for each region based on differences in income expressed as GDP per capita adjusted for purchasing power parity^52^.

Second, we estimated the market value of air quality improvements by assessing the associated impacts on productivity. The small particle size of PM_2.5_ allows the air pollutant to penetrate buildings and therefore affect a broad range of human activities. Based on a review of the empirical literature on the productivity impact of fine particulate matter in a range of sectors and regions (Supplementary Information, SI.5), we calibrated sector-specific exposure–response functions (for industry, services, and agriculture), reflecting that how air pollution impacts productivity depend on the type of work performed. We then used the sector-specific model of the whole economy JRC-GEM-E3^53,54^ to analyse how the changes in labour productivity inferred from the exposure–response functions would propagate through the economy, including national and international supply chains and trade. We expressed the economic impacts in terms of percentage impacts on global and regional GDP.

### Uncertainty analysis

We explicitly tracked the uncertainty related to the health impacts of air pollution and the valuation of those impacts (SI Datafile). In the health assessment, we accounted for epidemiological uncertainty by using the 95% confidence intervals of the relative risk factors that relate exposure to air pollution to disease risk^47^. In the economic valuation of changes in mortality, we accounted for uncertainty in both assessments. For the estimates of market impacts, we incorporated uncertainty intervals of productivity impacts derived from a dedicated literature survey (Supplementary Information, SI.5), and for the estimates of non-market impacts, we followed guidelines by the OECD^51^ and combined uncertainty in the base valuation of reduced mortality risk with uncertainty in the income elasticity used in the benefit transfer method (e.g., the low end of the estimates combines a low base valuation with a high-income elasticity).

In previous analyses, we also assessed the sensitivity of emissions estimates to different model specifications and found the current method to be the most robust^55,56^. As an additional sensitivity test, we perturbed ammonia emissions by 20% in either direction and found that the changes in premature mortality associated with the different diet scenarios were well within the epidemiological uncertainty range (SI Tables 23–25). Given our use of one particular agriculture-economic model, we were not able to quantify the uncertainty related to market feedback. We encourage the development of dedicated multi-model comparisons of global agriculture-economic models as a suitable way to quantify these uncertainties^27^.

### Reporting summary

Further information on research design is available in the Nature Portfolio Reporting Summary linked to this article.

### Supplementary information

Supplementary Information

Description of Additional Supplementary Files

Supplementary Data 1

Supplementary Data 2

Reporting Summary

## Data Availability

The data that support the findings of this study are available in the Supplementary Datafiles 1 and 2.

## Code availability

The code of the CAPRI, TM5-FASST, and GEM-E3 models are described in the Supplementary Information and the references cited therein. The models are maintained by the Joint Research Centre of the European Commission. Model codes are available upon request.
