# Supplementary material for: The global and regional air quality impacts of dietary change
Source: Nat Commun. 2023 Oct 6;14:6227. doi: 10.1038/s41467-023-41789-3 (PMC10558460; doi:10.1038/s41467-023-41789-3)
Supplement: Supplementary file 1 — Supplementary Information [file 41467_2023_41789_MOESM1_ESM.pdf]

The global and regional air quality impacts of dietary change

Supplementary Information

Marco Springmann[1,2]*, Rita Van Dingenen[3], Toon Vandyck[4,5], Catharina Latka[6], Peter Witzke[6], Adrian Leip[7]

[1]Environmental Change Institute, University of Oxford, Oxford, UK.
[2]Centre on Climate Change and Planetary Health, London School of Hygiene and Tropical Medicine, London, UK.
[3]European Commission, Joint Research Centre (JRC), Ispra, Italy.
[4]European Commission, Joint Research Centre (JRC), Seville, Spain.
[5]Department of Economics, KU Leuven, Leuven, Belgium
[6]Institute for Food and Resource Economics, University of Bonn, Germany.
[7]European Commission, DG Research & Innovation, Bioeconomy and Food Systems Unit, Brussels, Belgium.

*Correspondence to: marco.springmann@ouce.ox.ac.uk

Table of Contents

1. The CAPRI model 2
2. Diet scenarios 4
3. Agricultural emissions 5
4. Air pollution 10
5. Economic valuation 22
6. Supplementary results 25
Supplementary references 40

1. The CAPRI model

CAPRI is a comparative-static partial equilibrium agricultural sector model developed for policy and market assessments. The modelling system combines a global market model with a regional supply model for agricultural commodities that iterate to achieve a market equilibrium for product quantities and prices [1]. The supply module consists of non-linear programming models that combine Leontief technologies for the variable costs of production activities with non-linear cost functions which capture the effects that changes in labour and capital have on decision making by farmers. Each of these programming models represents an average aggregate farm which operates to maximize profit under constraints related to resources (land, nutrients), legislation (e.g. the EU's Common Agricultural Policy), and trade regulation [2]. Prices are exogenous to the supply module and provided by the market module. The global market module is a spatial, non-stochastic global multi-commodity model. It is defined by a system of behavioral equations representing agricultural supply, demand for food, feed and from processing industries, as well as multilateral trade relations differentiated by commodity and geographical units. National average food consumption is based on food availability data from FAOSTAT and Eurostat [3]. Consumer demand is elasticity-based and represented by indirect utility functions depending on prices and increasing in income. Bilateral trade and attached prices are captured using the Armington approach [4]. SI Table 1 lists the regional aggregation of the model.

CAPRI accounts for future development pathways. It is calibrated to the set of socio-economic scenarios developed by the impact assessment community, which consists of shared socio-economic pathways (with different projections of GDP changes and income) and representative concentration pathways (with different projections of temperature changes and precipitation) [5–8]. These pathways determine the alignment of the simulations with macro variables (population, GDP) and provide estimates of yield changes due to agronomic development and climate change linked to radiative forcing [9], whilst accounting for the (endogenous) implementation of mitigation technologies [10–12]. For our analysis, we focused on the year 2030 using a middle-of-the-road socio-economic development pathway (SSP2) and a representative concentration pathway with a moderate carbon price of 50 USD/tCO_2eq in 2030 (RCP6.0). For comparison, we also included a more optimistic pathway to 2050 with lower population growth and higher income growth (SSP1), together with greater mitigation efforts (RCP2.6) [7,8].

SI Table 1. List of countries and regions included in the CAPRI model.

Regional aggregate	CAPRI country/region
Europe	Belgium and Luxembourg
	Denmark
	Germany
	Greece
	Spain
	France
	Ireland
	Italy
	The Netherlands
	Austria
	Switzerland
	Portugal

	Sweden
	Finland
	United Kingdom
	Czech Republic
	Hungary
	Poland
	Slovenia
	Slovak Republic
	Estonia
	Lithuania
	Latvia
	Cyprus
	Malta
	Bulgaria
	Romania
	Norway
	Turkey
	Albania
	Serbia
	Montenegro
	Kosovo
	Croatia
	Bosnia and Herzegovina
	Ukraine
	Rest of Europe
Eurasia	Macedonia
	Russia
	Belarus
	Kazakhstan
	Former Soviet Union without Russia
Africa	Morocco
	Nigeria
	Ethiopia
	South Africa
	Tunisia
	Algeria
	Egypt
	African LDCs
	Africa Rest (practically ACP)
Middle East	Middle East
	Israel
Southern Asia	India
	Pakistan

	Bangladesh
Eastern Asia	China
	Taiwan
	South Korea
South-East Asia and developing Pacific	Malaysia
	Indonesia
	Thailand
	Vietnam
	Asian and Oceania LDC
	Rest of Asia
Asia-Pacific Developed	Japan
	Australia and New Zealand
North America	USA
	Canada
Latin America and Caribbean	Mexico
	Argentina
	Brazil
	Middle and South America ACP
	Rest of South and Middle America
	Venezuela
	Chile
	Uruguay
	Paraguay
	Bolivia

2. Diet scenarios

We used CAPRI to evaluate the changes in agricultural emissions associated with dietary changes towards a set of healthy and sustainable dietary patterns. The dietary patterns were based on those developed by the EAT-Lancet Commission on Healthy Diets from Sustainable Food Systems and included nutritionally balanced flexitarian (FLX) diets with low levels of animal source foods, as well as vegetarian (VEG) and vegan (VGN) diets in which meat or all animal source foods were replaced by a mix of legumes and fruits and vegetables [13]. The dietary patterns were regionalised, e.g. by preserving regional preferences for the type of grains and by implementing food-group recommendations as lower and upper values [14]. As a result, lower than maximum recommended intake (e.g. of red meat in the FLX scenario) was preserved, as was higher than minimum recommend intake (e.g. of fruits and vegetables in all scenarios). For the analysis, the diets are compared to a benchmark (BMK) diet that represents the baseline diet in this study. SI Table 12 provides an overview of the diet scenarios, aggregated to the detail with which food groups are represented in CAPRI.

3. Agricultural emissions

We calculated the agricultural emissions that are consistent with the diet scenarios in each region in two steps. First, we shifted CAPRI's baseline diets to an interim scenario that involved adjustments towards the scenario diets, in particular the flexitarian diet scenario as defined above. Following any "shock" to the baseline situation, the model adjusts agricultural supply chains, which impacts the relative prices of goods. The change in prices affects demand such that the final demand differs from the initial shocks. For complete alignment with the diet scenarios, we then scaled food demand and the associated emissions in a second step, for which we used the set of region and food-group specific emissions footprints (emissions per quantity of food demanded) from the first iteration. This scaling is a supplementary calculation that allows us to analyse scenarios that include substantial deviations from reference trajectories such as the diet scenarios considered for this study.

For the quantification of air pollutant emissions we made use of CAPRI's 'leakage module' and AgLink [2,15]. For European regions, CAPRI follows closely IPCC Tier 1 or 2 methodologies that link emission factors per activities (such as animal herds) to activities [16]. For non-European regions CAPRI covers produced quantities (such as pork) but not the underling activities (pig breeding and fattening). For these regions, the emission accounting in CAPRI relies on product-based emission factors that are estimated in two steps. The first is a technical conversion of activity-based emission factors in a linear programming step into product-based emission factors. Subsequently these preliminary estimates (priors) are adjusted in a second (Bayesian estimation) step so they are in line with time series of inventories by country and emission type reported by international agencies. The priors were adopted from AgLink and consider trends in emission intensities [17,18]. The method also produces more robust results than applying Tier 3 methods in CAPRI [19,20].

SI Table 2 shows product-based emissions factors estimated for this study for NH_3 and for methane (CH_4) in aggregate world regions. We aggregated items with similar emission coefficients (e.g., non-rice grains and fruits & vegetables) for ease of presentation. The emissions factors for methane and nitrous oxide (generated from volatilization of ammonia) are comparable in magnitude to those listed in the literature [21].

SI Table 2. Product-based emissions factors in 2030 by air pollutant, food group and world region (in kg of gas per metric ton of fresh matter of food).

	EU	non-EU Eurasia	Africa	Canada, US, Mexico	Other America	Asia and Pacific	World
				Ammonia [kg NH3 / t FM]			
rice	1.11	1.36	0.71	0.11	0.79	2.02	1.89
grains except rice	0.67	1.12	0.55	0.88	0.52	1.29	0.96
legumes	0.33	0.32	0.33	1.28	0.11	0.98	0.58
nuts & seeds	1.21	0.73	0.57	1.20	0.38	2.32	1.34
fruits & vegetables	0.12	0.11	0.12	0.11	0.12	0.12	0.12
roots & other	0.12	0.12	0.12	0.12	0.12	0.12	0.12
sugar	0.46	0.41	0.45	0.45	0.45	0.45	0.45
beef	36.52	38.27	207.77	59.31	115.87	151.58	110.62
lamb	22.28	158.89	284.45	353.76	450.30	192.26	214.82
pork	14.97	25.12	123.00	19.82	34.99	15.13	18.59
poultry	8.51	10.95	36.76	16.75	15.80	48.00	26.54
eggs	4.64	7.69	8.13	8.43	8.42	8.28	7.93
milk	2.52	4.58	12.95	2.10	5.26	5.23	4.91
				Methane [kg CH4 / t FM]			
rice	34.91	59.18	61.26	46.81	37.25	50.04	50.04
grains except rice	0.00	0.00	0.00	0.00	0.00	0.00	0.00
legumes	0.00	0.00	0.00	0.00	0.00	0.00	0.00
nuts & seeds	0.00	0.00	0.00	0.00	0.00	0.00	0.00
fruits & vegetables	0.00	0.00	0.00	0.00	0.00	0.00	0.00
roots & other	0.00	0.00	0.00	0.00	0.00	0.00	0.00
sugar	0.00	0.00	0.00	0.00	0.00	0.00	0.00
beef	338.89	289.18	1319.33	522.63	1275.04	1182.07	938.13
lamb	502.10	659.65	962.27	1304.18	1547.41	609.42	726.33
pork	33.17	35.16	73.71	70.75	30.91	41.29	43.03
poultry	0.51	12.49	11.43	25.55	2.57	8.23	10.88
eggs	0.41	1.25	1.44	1.52	1.55	1.41	1.33
milk	23.39	32.52	72.61	15.93	38.93	40.65	36.21

Below we detail the different steps of our simulation approach in more detail. SI Table 3 contains an explanation of the symbols used for this. We use the following abbreviations:

Abbreviations:

fg Food group

ssp *Shared* socio-economic pathway

y Year

r Region

step We quantified intake by food groups and corresponding agricultural emissions in two steps,

(i) Step = int: Interim simulation with partially adjusted diets. This interim run diet deviates from the target diets.

(ii) Step = *adj*: Both diets and emissions are scaled such that the adjusted diets equal the target diets (trg"). To reflect the fact that changes in consumption occur often in a different country than the changes in emission, we follow a global pool approach to calculate shifts in emission linked to consumption of imported foods. We assume here that the share of countries contributing to total global production is not changing by moving from a shocked to an adjusted situation.

SI Table 3. Overview of mathematical symbols used in the model description.

Symbol	*Unit*	*Description*
$IMPT$	Gg yr^{-1}	Imports of food group *fg* to region *r*
$PROD$	Gg yr^{-1}	Production of food group *fg* in region *r*
$FEED$	Gg yr^{-1}	Use of food group *fg* in region *r*
$HCOM$	Gg yr^{-1}	Human consumption of food group *fg* in region *r*
$TDMD$	Gg yr^{-1}	Total domestic demand for food group *fg* in region *r*, including demand for human consumption (intake and household food waste), feed, processing, biofuels, export and market losses
$\Delta INTK_{ssp,y,r,fg}$	g cap^{-1} day^{-1}	Difference of intake between the target diets and the diets under the interim CAPRI simulation in 'SSP' *ssp*, year *y*, region *r* and for food group *fg*
$E^{step}_{x,ssp,y,r,fg}$	Gg yr^{-1}	Emissions from commodity *fg* in region *r* for 'step' *step.* We consider emissions of NH_3 (measures in NH_3-N) and CH_4 (measured in CH_4).
$f^{int}_{IMPT,ssp,y,r,fg}$	dimensionless	Fraction of imported consumption of food group *fg* in region *r* relative to total domestic supply in the interim scenario
$INTK^{step}_{ssp,y,c,fg}$	g cap^{-1} day^{-1}	Intake of food group *fg* in region *r*, referring to the 'step' *step* as defined above.
$s_{INTK,ssp,y,r,fg}$	dimensionless	Scaling factor to be applied to the CAPRI interim diet to move to the intake of the healthy target diets or to the benchmark diet
$s^{LVST}_{INTK,ssp,y,GLOB,fg}$	dimensionless	Scaling factor for global consumption of livestock products (red meat, poultry meat and eggs, and milk and dairy products) for which CAPRI considers feed intake between the CAPRI interim intake and the target diets
$\Delta E_{ssp,yr,r,fg}$	Gg NH3-N yr^{-1}	Difference in emissions from the production of food group *fg* in region *r* between the CAPRI interim simulation and the target diets

$\Delta HCOM^{impt}_{ssp,yr,GLOB,fg}$	Gg yr^{-1}	Difference in imported food consumption of food group *fg* summed over all regions *r* between the CAPRI interim simulation and the target diets
$\Delta INTK_{ssp,yr,r,fg}$	g cap^{-1} day^{-1}	Difference in daily intake of food group *fg* in region *r* between the CAPRI interim simulation and the target diets
$r^{dom}_{ssp,yr,r,fg}$	dimensionless	Changes in consumption of domestically produced food products relative to total demand for food group *fg* in region *r.*
$r^{feed}_{ssp,yr,r,fg}$	dimensionless	Change in demand of marketable feed relative to total domestic demand for feed product *fg* in region *r.*
$r^{impt}_{ssp,yr,GLOB,fg}$	dimensionless	Change in consumption of imported food products relative to global total food production for food group *fg*
$r^{total}_{ssp,yr,r,fg}$	dimensionless	Total changes in production of food group *fg* in region *r* when moving from the CAPRI interim simulation to the target diets, relative to total food production for food group *fg.*

Details of the model simulation

Adjusting emissions to reflect intake of healthy diets:
The targeted intake of foods in the diet scenario is composed of the intake that results from the interim scenario plus a scaling factor.

$$INTK^{trg}_{ssp,y,r,fg} = INTK^{int}_{ssp,y,r,fg} + \Delta INTK_{ssp,y,r,fg} \quad \text{Equation 1}$$

We calculate a scaling that is applied to subsequent model parameters.

$$s_{INTK,ssp,y,r,fg} = \frac{INTK^{trg}_{ssp,y,r,fg}}{INTK^{int}_{ssp,y,r,fg}} \quad \text{Equation 2}$$

Domestically produced food:
We account for the fact that changes in production (and thus emissions) occur partly in countries different from those where the consumption is adjusted. For all food groups we calculate in a first step the fraction of food that is supplied through imports. This allows us to separately estimate domestic emissions and emissions in the countries of origin of imports.

$$f^{int}_{IMPT,ssp,y,r,fg} = \frac{IMPT}{IMPT+PROD}\bigg|^{int}_{ssp,yr,r,fg} \quad \text{Equation 3}$$

Changes in emissions due to changes in consumption of domestically produced food are captured by the change in consumption from domestic sales $r^{dom}_{ssp,y,r,fg}$relative to total domestic supply and calculated as follows:

$$r^{dom}_{ssp,y,r,fg} = s_{INTK,ssp,y,r,fg} \cdot \frac{HCOM}{TDMD}\bigg|^{int}_{ssp,yr,r,fg} \quad \text{Equation 4}$$

where $HCOM$ is total human consumption (intake and food waste in households) and $TDMD$ is total demand for human consumption, feed, processing including biofuels, and export.

Imported food:
For changes in food consumption from imported food we apply a ‘global pool market’ approach. This approach is also used in the CAPRI LCA (Life Cycle Assessment) module for consumption-based commodity emission factors [22,23]. It recognizes that the global market is fluid and changes in bi-lateral trade flows are likely to have further knock-on effects influencing the GHG intensity of imported feed stuff. All changes in imports are therefore summed up globally ($\Delta HCOM^{impt}_{ssp,yr,GLOB,fg}$, see Eqn. 5) and used for calculating the change in consumption of imported food globally relative to total global production ($r^{impt}_{ssp,yr,GLOB,fg}$, see Eqn. 6). Ignoring economic feedback effects when moving from the interim to the adjusted status (as in the scaling of the domestic food component), the production of the food group in each country is scaled using the same factor (see Eqn. 7).

$$\Delta HCOM^{impt}_{ssp,yr,GLOB,fg} = \sum_r \left\{ f^{int}_{IMPT,ssp,y,r,fg} \cdot s_{INTK,ssp,y,r,fg} \cdot HCOM_{ssp,y,r,fg} \right\} \qquad \text{Equation 5}$$

$$r^{impt}_{ssp,yr,GLOB,fg} = \frac{\Delta \mathrm{HCOM}^{impt}_{ssp,yr,GLOB,fg}}{\sum_r PROD_{ssp,y,r,fg}} \qquad \text{Equation 6}$$

$$r^{impt}_{ssp,yr,r,fg} = r^{impt}_{ssp,yr,GLOB,fg} \qquad \text{Equation 7}$$

Marketable feed:
The CAPRI commodity-based emission factors do not include emissions from feed production if the feed is a ‘commodity’ in the sense that it is traded and also used for human consumption directly or has other uses. This ensures that the emission factors remain ‘production based’ and hence allocated to the country of feed production. Changes in consumption of livestock products will trigger changes in the production of marketable feed. To capture this effect, we calculated the change in total consumption of livestock products, aggregating over different livestock products on the basis of their energy content:

$$s^{LVST}_{INTK,ssp,yr,GLOB,fg} = \frac{\sum_r INTK^{adj}_{ssp,y,r,fg}\Big|_{fg\in\{rmea,poul,milk\}}}{\sum_r INTK^{int}_{ssp,y,r,fg}\Big|_{fg\in\{rmea,poul,milk\}}} \qquad \text{Equation 8}$$

The total production of marketable feed changes proportionally to the consumption of livestock products:

$$r^{feed}_{ssp,y,r,fg} = \left(s^{LVST}_{INTK,ssp,y,r,GLOB,fg} - 1\right) \cdot \frac{FEED}{TDMD}\Big|^{int}_{ssp,yr,r,fg} \qquad \text{Equation 9}$$

Total effect on emissions:
The total effect on production and thus emission is obtained from the sum of the effects caused by changes of domestically produced food, imported food, and feed:

$$r^{total}_{ssp,y,r,fg} = r^{dom}_{ssp,y,r,fg} + r^{impt}_{ssp,y,r,fg} + r^{feed}_{ssp,y,r,fg} \qquad \text{Equation 10}$$

$$E^{adj}_{ssp,y,r,fg} = E^{int}_{ssp,y,r,fg} \cdot \left(1 + r^{total}_{ssp,y,r,fg}\right) \qquad \text{Equation 11}$$

4. Air pollution

Reduced NH_3 emissions from dietary changes towards more plant-based and low-nitrogen diets are expected to reduce the share of ammonium nitrate and sulfate in fine particulate matter (PM2.5). Low-nitrogen diets also reduce methane emissions, which can add to the air quality benefit via the impact on global background ozone [24,25].

TM5-FASST:

We use the reduced-form TM5-FASST air quality model [26] to compute global PM2.5 and ozone concentration grid maps for the selected scenarios of dietary change. TM5-FASST is built on linearized region-to-grid emission-concentration response fields that have been precomputed with the TM5 2-way nested global Chemistry-Transport Model [27], based on a 20% emission perturbation of a reference pollutant emission set (RCP year 2000) in each of 56 defined source regions. 'Region-to-grid' means that emissions are given as model input as regional totals (with implied and fixed spatial distribution for each source region) while the resulting pollutant concentrations are obtained as 1°x1° resolution grid maps.

For each 1°x1° grid cell, the change in concentration of component *j* in receptor *y* resulting from a -20% emission perturbation of precursor *i* in all grid cells of source region *x*, is expressed by a unique source-receptor (SR) coefficient $A_{ij}[x, y]$:

$$A_{ij}[x, y] = \frac{\Delta C_j(y)}{\Delta E_i(x)} \text{ with } \Delta E_i(x) = 0.2 E_{i,\mathrm{ref}}(x) \qquad \text{Equation 12}$$

The total concentration of a component (or metric) *j* in receptor region *y*, resulting from emissions of *all* n_i precursors *i* at *all* n_x source regions *x,* is obtained as a 'perturbation' on the reference-simulation concentration, by summing up all the respective SR coefficients scaled with the actual emission 'perturbation', being the difference between the reference and actual emission:

$$C_j(y) = C_{j,ref}(y) + \Delta C_j(y) \qquad \text{Equation 13}$$

$$\Delta C_j(y) = \sum_{k=1}^{n_x} \sum_{i=1}^{n_i} A_{ij}[x_k, y] \cdot [E_i(x_k) - E_{i,\mathrm{ref}}(x_k)] \qquad \text{Equation 14}$$

$C_{j,ref}(y)$, $A_{ij}[x_k, y]$ and $E_{i,\mathrm{ref}}(x_k)$ are fixed and have been determined 'once and for all' using the RCP year 2000 reference TM5 run and the 20% emission perturbations on the latter. The only scenario-dependent input is $E_i(x_k)$, i.e. the actual scenario emission for the considered pollutant, aggregated over the source region. Pollutants C_j include particulate matter components (SO_4, NO_3, NH_4, BC, particulate organic matter – POM), trace gases (SO_2, NO, NO_2, NH_3, O_3), and deposition fluxes of BC, N and S species. In the case of ozone, the n_i precursors in the above equation comprise [NO_x, NMVOC, CO, CH_4].

Non-linearity correction for ammonium and nitrate

Van Dingenen et al [26] found that the linear relationship between emissions and PM2.5 (Eq. 13) is sufficient for small emission perturbations, but that the relationship exhibits increasingly non-linear behaviour for larger perturbations, particularly for the formation of secondary PM2.5 (ammonium salts) from NO_x, NH_3 and SO_2. This is relevant in the frame of this study for year 2030 where for developed countries programmed air quality measures strongly reduce emissions of NO_x below levels of the reference year 2000, and in particular for the USA and Europe where the considered scenarios are introducing additional medium to strong NH_3 reductions. As documented in previous

studies, when NO_x becomes the limiting factor for nitrate formation, the response of ammonium nitrate formation to NH_3 emission changes is lower than in a regime with simultaneous availability of both NH_3 and NO_x while the response to NO_x emission changes becomes stronger [28–30].

To address these secondary non-linearity effects in the TM5-FASST tool, we introduce a correction factor *f*(MR) for the formation of NO_3 + NH_4 where MR represents the emission molar ratio of the components, as an emissions-based proxy-metric for the chemical regime in which the formation of ammonium salts takes place:

$$MR = \frac{NH_3/17}{NO_x/46 + SO_2/64} \qquad \text{Equation 15}$$

The corrected concentration change of NH_4+NO_3 from changes in NO_x, NH_3 and SO_2 emissions, relative to the reference scenario is obtained from:

$$\Delta C_{scen}^{corr} = f_{NH3}(MR) \times \Delta C_{scen}^{FASST}(NH_3) + f_{NOx}(MR) \times \Delta C_{scen}^{FASST}(NO_x) + f_{SO2}(MR) \times \Delta C_{scen}^{FASST}(SO_2)$$

Where ΔC_{scen}^{FASST} is the standard TM5-FASST linear computation as in Eq. 14 for precursors NH_3, NO_x and SO_2 respectively, computed from the evaluated emissions magnitudes, while the correction factor *f* depends on the emission molar ratio of the precursors NH_3, NO_x and SO_2 (Eq. 15).

The analysis of the SO_4 response to NH_3, NO_x and SO_2 emission perturbations outside the standard -20% range shows that it is not significantly affected by non-linearity effects, therefore the 'standard' TM5-FASST approach, based on a -20% perturbation response sensitivity, is deemed adequate in the context of the present study.

f(MR) values are obtained from a set of runs with the full TM5 model where, additional to the standard 20% emission perturbation run (Eq. 12), source-receptor coefficients were determined for emission changes between -80% to +100% for NO_x, SO_2 and NH_3. These additional perturbations encompass a wide range of emission MRs and are available for selected relevant source regions: Europe, USA, Japan, China+, India+ [26].

$$f_i[MR(p)] = A_{i,p}[x]/A_{i,0.8}[x] \qquad \text{Equation 16}$$

$A_{ij,0.8}[x]$ and $A_{ij,p}[x]$ are the source-receptor coefficients within the source region *x* itself, for the standard 20% perturbation and larger perturbations *p* respectively, applied on NH_3, NO_x and SO_2 separately. $MR(p)$ is the emission molar ratio corresponding to each of the respective emission perturbations. Trends of *f*(MR) for the selected regions are shown in SI Figure 1. Trend lines are well represented by the ad-hoc chosen functional shape:

$$f(MR) = 1 + b \ln\left(\frac{MR}{MR_{0.8Ref}}\right) \qquad \text{Equation 17}$$

which ensures that the non-linearity correction factor *f* returns a value of 1 when the scenario MR equals the MR of the -20% perturbation of the reference case ($MR_{0.8Ref}$). Parameter *b* is obtained from least-squares fitting. Values for $MR_{0.8Ref}$ and *b* are given in SI Table 4.

SI Table 4. Parameters for the logarithmic function describing the non-linearity correction factor for NH_3, NO_x and SO_2 emissions in terms of emission molar ratio, reference emission molar ratio at 20% emission reduction $MR_{0.8Ref}$ and fit parameter *b* (Eq. 17).

**NH_3**	$MR_{0.8Ref} = \frac{0.8NH_{3\text{Ref}}/17}{NO_{x\text{Ref}}/46 + SO_{2\text{Ref}}/64}$	
**TM5-FASST Source Region**	$\boldsymbol{MR_{0.8Ref}}$	***b***
China+	0.57	-0.31
Europe	0.56	-0.30
Japan	0.25	-0.19
India+	0.94	-0.40
USA	0.25	-0.12
**NO_x**	$MR_{0.8Ref} = \frac{NH_{3\text{Ref}}/17}{0.8NO_{x\text{Ref}}/46 + SO_{2\text{Ref}}/64}$	
**TM5-FASST Source Region**	$\boldsymbol{MR_{0.8Ref}}$	***b***
China+	0.78	0.51
Europe	0.79	0.51
Japan	0.37	0.57
India+	1.33	-0.48
USA	0.36	0.96
SO_2	$MR_{0.8Ref} = \frac{NH_{3\text{Ref}}/17}{NO_{x\text{Ref}}/46 + 0.8SO_{2\text{Ref}}/64}$	
**TM5-FASST Source Region**	$\boldsymbol{MR_{0.8Ref}}$	***b***
China+	0.79	0.67
Europe	0.76	0.59
Japan	0.33	0.68
India+	1.29	0.26
USA	0.34	1.09

SI Figure 1. Response correction factor on linear TM5-FASST source-receptor coefficients for NH_4+NO_3 as a function of emission molar ratios corresponding to emission perturbations of NH_3 (blue), NO_x (orange) and SO_2 (grey) in the range [0.2, 2.0]xE_{ref} for the 6 regions evaluated. Intermediate points for NO_x are additional perturbation experiments at 0.5 and 1.5xE_{ref}. The central dots with correction factor = 1 correspond to the 'standard' 0.8xE_{ref} perturbation experiment. For Germany, the SO_2 perturbations are not available. Dotted lines show best-fit functions (Eq. 17, SI Table 4)

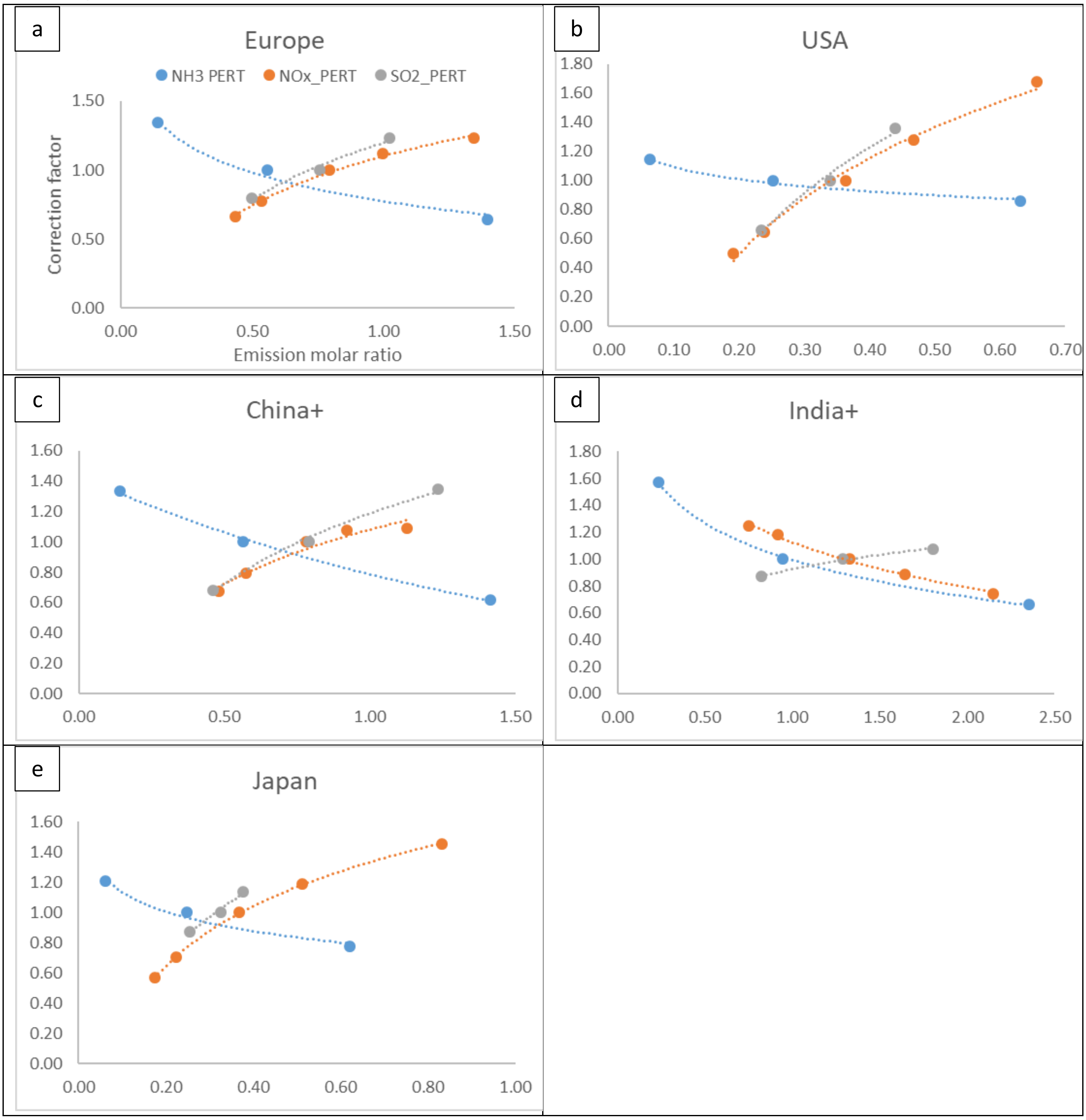

The trends shown in SI Figure 1 imply that:

- The response to NH_3 emissions when MR is lower (i.e. more NH_3-limited) than the -20% perturbation of the reference case leads to a correction factor > 1 (blue line in SI Figure 1). The corrected response to NH_3 emission reductions will therefore be larger than with the standard linearized TM5-FASST computation, i.e. the uncorrected TM5-FASST approach for the resulting secondary PM2.5 is biased high for NH_3 emission reductions. The actual response to NH3 emission increase is overestimated in the standard FASST approach (correction factor <1), hence also here the standard approach leads to a positive bias in the resulting PM2.5.

- A reduction in NO_x and SO_2 emissions under unaltered NH_3 emissions leads to an increase in MR. For all regions, except for NO_x in India, increasing MR leads to a correction factor > 1 on the linearized TM5-FASST response to NO_x and SO_2 emission changes (orange and grey lines in SI Figure 1). Also here the uncorrected TM5-FASST approach is biased high towards NO_x and SO_2 emission reductions. Similarly, as a correction factor < 1 results for an emission increase (MR decrease), also in this case the standard approach returns a positive bias in resulting PM2.5.

- <u>Simultaneous</u> changes in NO_x, NH_3 and SO_2 emissions may lead to a MR at either side of the reference value, depending on the relative magnitudes of the emission changes. For a given MR, the response correction factors to apply depend on the resulting MR value, and will be (except for India) either a combination of (> 1 for NO_x and SO_2, and <1 for NH_3), or (<1 for NO_x and SO_2 and > 1 for NH_3).

In the present study we apply the correction factors from the function in Eq. 17, using the regional MRs corresponding to each of the scenarios. We recall that the NO_x emission trends in the dietary change scenarios have been copied from SSP2, projecting substantial NO_x mitigation by 2030 in developed economies (Europe, North America, Japan) compared to the TM5-FASST reference set of year 2000, while NH_3 emissions are modelled with CAPRI.

A possible issue with the fitted trend line is the extrapolation to MR values outside the fitted range. SI Table 5 shows the range of MRs obtained from the perturbation studies, separately for the benchmark and dietary change scenarios, together with the range observed in the evaluated scenarios in this study. At the low side (determined by low NH_3 emissions in the most stringent dietary change cases) there are no issues with extrapolation beyond the fitted function range. At the high side (driven by strong NOx emission reductions in the benchmark scenarios for 2030 and 2050), the highest scenario MRs of all observed regions (except India) extend beyond the highest MR found in the perturbation studies.

We therefore validate the correction function against the full TM5 model outcome for a stringent emission reduction scenario (low NOx, hence high MR relative to reference). More details on the selected scenario (Global Energy Assessment MIT2030 scenario, Rao et al [31]) are given by Van Dingenen et al [26]. Relevant regions with strong NO_x reductions are Europe, USA, and Japan, while the China and India regions are more affected by SO_2 reductions. The NO_x and SO_2 reductions in this scenario lead to high MR values in selected regions, well extending beyond the MRs from the perturbation studies (SI Table 5). SI Figure 2 shows the 'standard' non-corrected TM5-FASST outcome for NO_3+NH_4, as well as the corrected outcome using Eq. 17, against the full TM5 model results. These results provide additional confidence in the selected ad-hoc correction function shape for strong reductions in NO_x. Only for China, the SO_2-driven correction on NO3 and NH4 formation deviates further from the TM5 result than the non-corrected one.

SI Table 5. Range of emission molar ratios for relevant regions across the extended perturbation studies, across the dietary scenarios evaluated in this study, and for the Global Energy Assessment (GEA) validation scenario. Values are aligned with the main driving precursor(s) in the respective cases. Current scenario values in bold are outside the MR range evaluated in the perturbation experiments. The applicability of the non-linearity correction function is justified by validation against the full TM5 model applying the correction on the GEA low-emission scenario (last column) – shown in SI Figure 2.

	Perturbation runs			Current scenarios (Benchmark)		Current scenarios (Diets)		GEA MIT2030
	Precursor	MRmin	MRmax	MRmin	MRmax	MRmin	MRmax	MR
China+	NH_3	0.1	1.4			0.2	1.0	
	NO_x	0.5	1.1	0.5	**1.9**			
	SO_2	0.5	1.2	0.5	**1.9**			1.8
Europe	NH_3	0.1	1.4			0.2	1.2	
	NO_x	0.4	1.3	0.8	**2.4**			4.5
	SO_2	0.5	1.0					
Japan	NH_3	0.1	0.6			0.1	0.6	
	NO_x	0.2	0.8	0.3	**1.4**			2.2
	SO_2	0.3	0.4					
India+	NH_3	0.2	2.4			0.3	0.9	
	NO_x	0.8	2.2	0.6	0.9			
	SO_2	0.8	1.8	0.6	0.9			1.7
USA	NH_3	0.1	0.6			0.4	**1.7**	
	NO_x	0.2	0.7	0.6	**3.5**			1.8
	SO_2	0.2	0.4					

SI Figure 2. Validation of the applied non-linearity correction for NO_3 + NH_4, comparing the standard and corrected TM5-FASST approach with the original full TM5 outcome for the MIT2030 Global Energy Assessment scenario with strong NO_x emission reductions in Europe, USA, Japan (left panel), and strong SO_2 emission reductions in China and India (right panel) For more details on the scenario we refer to Van Dingenen et al [26].

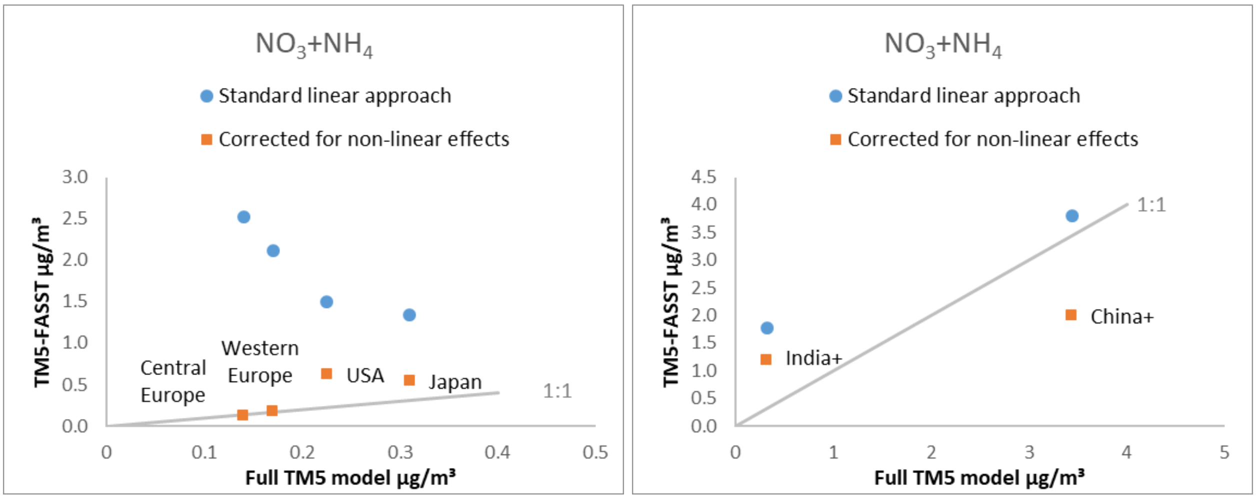

The obtained correction functions were implemented in the TM5-FASST model using the respective function parameters for the regions in SI Table 4. For European TM5-FASST regions/countries, the function parameters for the European region were applied individually. For regions where no correction functions could be fitted, proxy regions were selected pro-forma as shown in SI Table 6. However, because regions outside the fitted relevant set are barely affected by the dietary changes in our scenarios, the choice of the non-linearity correction function has no impact on the calculated benefits from dietary change.

SI Table 6: Selection of proxy region for non-linearity correction function for regions where the fitting is not available.

**FASST emission region**	**Correction function proxy region**
EU countries, UK, Western Balkan	Europe
Korea	Japan
Rest of South and South-East Asia	India
Africa	India
Rest of the World	Europe

Mapping of CAPRI regions to TM5-FASST regions

For the analysis, we mapped the changes in ammonia and methane emissions from CAPRI to the resolution of TM5-FASST and gap-filled other sectoral emissions by using the emissions data from the Climate Model Intercomparison Project (CMIP6 [32]) that matched the set of socio-economic and emissions pathways we used in CAPRI. SI Tables 7-8 provide an overview of baselines emission for the air pollutants focused on in this study (NH_3, CH_4). The tables compare the estimates of air-pollutant emissions for the baseline SSP2-4.5 runs (i.e. $E_{i,\mathrm{ref}}(x_k)$ in Eq. 14) and the gap-filled CAPRI estimates (i.e. $E_i(x_k)$ in Eq. 14).

SI Table 7. Comparison of 2010 emissions baselines for NH_3 between SSP2-4.5 and CAPRI.

Region	Agricultural NH_3 emissions (Tg/yr)		Total NH_3 emissions (Tg/yr)	
	SSP2	CAPRI	SSP2	CAPRI
Africa	3.2	5.8	8.5	11.1
Asia-Pacific Developed	1.2	1.5	1.4	1.7
Eastern Asia	10.2	8.3	12.9	11
Eurasia	1.5	2.3	2.3	3.1
Europe	4.5	3.9	5	4.4
Latin America and Caribbean	5.7	6.6	8.4	9.3
Middle East	0.9	0.6	1.3	1
North America	4	4.3	4.8	5.1
South-East Asia and developing Pacific	3.1	3	4.9	4.9
Southern Asia	9.8	6.5	13.1	9.8
**Global**	**44**	**42.7**	**62.5**	**61.2**

SI Table 8. Comparison of 2010 emissions baselines for CH_4 between SSP2 and CAPRI.

Region	Agricultural CH_4 emissions (Tg/yr)		Total CH_4 emissions (Tg/yr)	
	SSP2	CAPRI	SSP2	CAPRI
Africa	14.5	14.4	44.8	45
Asia-Pacific Developed	4.5	3.7	7.4	6.6
Eastern Asia	17.2	16.3	71.2	70.8
Eurasia	4.8	3.8	38.1	37.6
Europe	11	10.7	23.4	22.5
Latin America and Caribbean	26.8	26.2	60.2	59.5
Middle East	1.6	0.03	18	17
North America	10.8	9.2	34.3	32.7
South-East Asia and developing Pacific	13.4	16.9	28.8	30.7
Southern Asia	27.8	25.4	45.4	43.2
**Global**	**133**	**127**	**372**	**366**

For mapping the CAPRI regions to those of TM5-FASST we used sectoral RCP pollutant gridded emission data sets (available from http://www.iiasa.ac.at/web-apps/tnt/RcpDb/) to estimate individual countries emissions belonging to an aggregated CAPRI region $REG_i=\{CNTR_1,\dots,CNTR_n\}$:

$$E_k^{CAPRI} = \frac{E_k^{RCP}}{\sum_n E_n^{RCP}} E_{REGi}^{CAPRI}$$

Where E_k^{CAPRI} is the estimated $CNTR_k$'s emission strength consistent with CAPRI, $E_k^{RCP}$is the sum over RCP grid cell emissions for country $CNTR_k$ in the agricultural sector, $\sum_n E_n^{RCP}$ is the sum over RCP grid cell emissions for region REG_i and E_{REGi}^{CAPRI} is the regionally aggregated emission from the CAPRI model. The individual country emissions are then re-aggregated to their respective TM5-FASST regions and used as input to the TM5-FASST tool. SI Table 9 provides an overview of the mapping of regions between CAPRI and TM5-FASST.

SI Table 9. Mapping of countries between CAPRI and TM5-FASST.

CAPRI REGION	COUNTRY NAME	FASST REGION
AFR_LDC	Benin	WAF
	Burkina Faso	WAF
	Burundi	EAF
	Central African Republic	EAF
	Chad	EAF
	Congo	WAF
	Democratic Republic of the Congo	EAF
	Eritrea	EAF
	Gambia	WAF
	Ghana	WAF
	Guinea	WAF
	Guinea-Bissau	WAF
	Kenya	EAF

	Liberia	WAF
	Madagascar	EAF
	Malawi	SAF
	Mali	WAF
	Mauritania	WAF
	Mozambique	SAF
	Niger	WAF
	Rwanda	EAF
	Senegal	WAF
	Sierra Leone	WAF
	Togo	WAF
	Uganda	EAF
	United Republic of Tanzania	EAF
	Zambia	SAF
	Zimbabwe	SAF
AFR_REST	Angola	SAF
	Botswana	SAF
	Cameroon	WAF
	Côte d'Ivoire	WAF
	Equatorial Guinea	WAF
	Gabon	WAF
	Lesotho	RSA
	Namibia	SAF
	Other Atlantic Ocean	WAF
	Other Indian Ocean	RSAS
	Swaziland	RSA
AL000000	Albania	RCEU
ALG	Algeria	NOA
ANZ	Australia	AUS
	New Zealand	NZL
ARG	Argentina	ARG
ASOCE_LDC	Cambodia	RSEA
	Democratic People's Republic of Korea	MON
	Lao People's Democratic Republic	RSEA
	Myanmar	RSEA
	Nepal	RSAS
	Solomon Islands	PAC
ASOCE_REST	Bhutan	RSAS
	Fiji	PAC
	Mongolia	MON
	Other Pacific Ocean	PAC
	Other Southeast Asia	RSEA
	Papua New Guinea	PAC
	Philippines	PHL
	Sri Lanka	NDE

	Timor-Leste	IND
	Vanuatu	PAC
AT000000	Austria	AUT
BA000000	Bosnia Herzegowina	RCEU
BEL	Belarus	UKR
BG000000	Bulgaria	BGR
BGD	Bangladesh	RSAS
BL000000	Belgium and Luxembourg	BLX
BOL	Bolivia	RSAM
BRA	Brazil	BRA
CAN	Canada	CAN
CH	Switzerland	CHE
CHL	Chile	CHL
CHN	China	CHN
CS000000	Serbia	RCEU
CY000000	Cyprus	GRC
CZ000000	Czech Republic	RCZ
DE000000	Germany	RFA
DK000000	Denmark	SWE
EE000000	Estonia	POL
EGY	Egypt	EGY
EL000000	Greece	GRC
ES000000	Spain	ESP
ETH	Ethiopia	EAF
FI000000	Finland	FIN
FR000000	France	FRA
FSU	Armenia	RUS
	Azerbaijan	RUS
	Georgia	RUS
	Moldova	UKR
	Tajikistan	RIS
	Turkmenistan	RIS
	Uzbekistan	RIS
	Kyrgyzstan	RIS
HR000000	Croatia	RCEU
HU000000	Hungary	HUN
IND	India	NDE
INDO	Indonesia	IDN
IR000000	Ireland	GBR
ISR	Israel	MEME
IT000000	Italy	ITA
JAP	Japan	JPN
KAZ	Kazakhstan	KAZ
KO000000	Kosovo	RCEU
LT000000	Lithuania	POL

LV000000	Latvia	POL
MAL	Malaysia	MYS
MEX	Mexico	MEX
MIDEAST	Afghanistan	RSAS
	Djibouti	EAF
	Iran	GOLF
	Iraq	GOLF
	Jordan	MEME
	Lebanon	MEME
	Libya	NOA
	Rest of Arab Peninsula	GOLF
	Saudi Arabia	GOLF
	Somalia	EAF
	Southern Sudan	EAF
	State of Palestine	MEME
	Sudan	EAF
	Syrian Arab Republic	MEME
	Yemen	GOLF
MK000000	Macedonia	RCEU
MO000000	Montenegro	RCEU
MOR	Morocco	NOA
MSA_ACP	Belize	RCAM
	Cuba	RCAM
	Dominican Republic	RCAM
	Guyanas South America	RCAM
	Haiti	RCAM
	Jamaica	RCAM
	Other Caribbean	RCAM
MT000000	Malta	ITA
NGA	Nigeria	WAF
NL000000	Netherlands	BLX
NO000000	Norway	NOR
PAK	Pakistan	RSAS
PAR	Paraguay	RSAM
PL000000	Poland	POL
PT000000	Portugal	ESP
REU	Rest of Europe	NOR
RO000000	Romania	ROM
RSA	Colombia	RSAM
	Costa Rica	RCAM
	Ecuador	RSAM
	El Salvador	RCAM
	Guatemala	RCAM
	Honduras	RCAM
	Nicaragua	RCAM

	Panama	RCAM
	Peru	RSAM
RUS	Russia	RUS
SE000000	Sweden	SWE
SI000000	Slovenia	AUT
SK000000	Slovakia	RCZ
SKOR	South Korea	COR
TAW	Taiwan	TWN
THAI	Thailand	THA
TUN	TUNESIA	NOA
TUR	Turkey	TUR
UK000000	United Kingdom	GBR
UKR	Ukraine	UKR
URU	Uruguay	ARG
USA	USA	USA
VEN	Venezuela	RSAM
VIET	VietNam	VNM
ZAF	South Africa	RSA

Exposure and health impacts

The relevant PM2.5 exposure metric is the annual mean ambient PM2.5 concentration, while the long-term O_3 exposure metric is the seasonal (6-month period with highest ozone) 8h-daily maximum ozone concentration. The native 1°x1° output resolution is regridded to 7.5'x7.5' and overlaid with respective SSP (year 2030) population grid maps of the same resolution [33] to evaluate population exposure at each grid cell. Premature mortalities are evaluated for 6 causes of death (COD) at each individual grid cell using the Global Burden of Disease (GBD) 2017 methodology for ambient air pollution [34–36]. Country-specific attributable premature mortalities are then obtained as the sum over the country's *n* grid cells.

$$M_{ref}(COD) = y_0(COD) \times \sum_{k=1}^{n} AF(COD, C_k) \times POP_k$$

where

C_k = the PM2.5 or O_3 exposure metric (concentration)

$Y_0(COD)$ = Country's baseline mortality rate for COD

POP_k = the exposed population in grid cell k

$AF(COD, C_k) = \frac{RR(COD,C_k)-1}{RR(COD,C_k)}$, the attributable fraction

$RR(COD, C_k)$ = the relative risk for COD at exposure level C_k

Following the GBD 2017 approach, ozone is associated with chronic obstructive pulmonary disease (COPD) all-age mortality, with a RR of 1.06, 95% C.I. (1.02, 1.10), per 10 ppb of ozone exposure and a theoretical minimum risk exposure level of 29.1 ppb.

Long-term exposure to PM2.5 is considered as a risk factor to 6 causes of death (COD): chronic obstructive pulmonary disease (COPD), lower respiratory infections (LRI), lung cancer (LC), ischemic heart disease (IHD), stroke and diabetes mellitus type 2 (DMT2). The relative risks (*RR*) for PM2.5 exposure are calculated from the integrated exposure-response (IER) functions developed by Burnett et al. (2014) and first applied in the GBD2010 study (Lim et al., 2012). The present study uses the latest updated function parameters including a theoretical minimum risk exposure level *zcf* uniformly distributed between 2.4 and 5.9 µg m^{-3} [36]. The RRs for IHD and stroke are age-specific (available at 5-years interval classes for 25 years and older) while the other CODs have a uniform *RR* distribution over all ages. The countries' baseline age-specific mortality rates were obtained from GBD 2017 [37], and projected mortalities (up till 2040) from the GBD 2016 Foresight project [38].

With respect to assessing exposure (i.e. overlaying pollutant concentration maps with population maps) we note that in the air-quality model TM5-FASST, spatial patterns in the concentration of air pollutants resulting from emissions within each region are fixed at 1°x1° resolution, and their magnitude is modulated according to the strength of emissions in the source region. This implicitly assumes that future changes in emissions from agriculture are happening where current emissions are already taking place, which generally is an acceptable assumption and supported by validation studies [39]. In particular, the secondary nature (and hence the longer lifetime and further atmospheric transport away from the sources) of PM2.5 from NH_3 emissions, and O_3 from methane makes the impact less sensitive to the spatial distribution of the emissions, and to the population distribution in comparison to direct PM2.5 emissions from transport and industry.

Regarding the impact of the relatively coarse model resolution, we acknowledge that in general, coarser model resolution can lead to a downward bias in mortality estimates compared to high resolution models. A modelling study for the US across different model resolutions conducted by Punger and West [40] indicated an underestimation of about 6% of mortality from ammonium nitrate exposure, and 3% for ammonium sulfate at 96km x 96km model resolution, compared to a 12km x 12km resolution. For comparison, for primary emitted PM2.5, the resolution bias is more than -20%. However, for our specific study, the impact of exposure to primary PM2.5 (and the associated bias) is cancelled out when evaluating differences between the CAPRI scenarios of the same year which are affected by NH_3 emission changes only.

5. Economic valuation

To estimate the economic benefits of improvements in air quality, we considered both market and non-market aspects.

For the non-market valuation of reduced mortality risk, our approach followed methods commonly applied in cost-benefit analysis [41]. In particular, we calculated the Value of Reduced Mortality Risk (VRMR) for country i in year t with the following equation

$$VRMR_i^t = VRMR_{USA}^{2015}\left(\frac{I_i^t}{I_{USA}^{2015}}\right)^{\varepsilon}$$

in which we set $VRMR_{USA}^{2015}$ equal to 5.2 (2.6-7.8) million USD. We choose the income elasticity ε equal to 0.8 (1.0-0.4) and we use GDP per capita in PPP terms as a measure of income I_i^t. We derive a range of results to reflect the uncertainty in the estimates, represented in a low, medium

and high sensitivity case. The low end of the estimates combines the lower bound health estimate with a low base $VRMR_{USA}^{2015}$ (2.6 million USD) and a high elasticity (of unity).

For the market valuation, we focused on changes in labour productivity that are related to air quality. For that purpose, we reviewed the recent empirical literature on productivity impacts of air pollution and, based on that, constructed three sets of productivity impact parameters, representing low, medium, and high impacts. We allowed productivity impacts to differ across three broad sectors, reflecting that air pollution impacts on productivity can depend on the type of work performed: industry, services and agriculture.

Case studies included in calibration of labour-market impacts

We used evidence from nine studies [42–50] on the productivity impact of fine particulate matter in a range of sectors in Europe, Asia and the US (SI Table 10). Evidence of air pollution impacts on the performance of soccer players [51], drivers [52], marathon runners [53], baseball umpires [54], public speakers [55], and chess players [56] are not considered here. These particular cases, although individually of great interest, are unlikely to offer a good basis to generalise findings to a broader sectoral and geographical scope. We furthermore ignore impacts on subjective well-being [57], sleep deprivation [58], and crime [59–61]. We limit our assessment of economic impacts to fine particulate matter (and one study for ozone, [48]), although we acknowledge that earlier studies have also found impacts of coarse particulate matter [62] and SO_2 [63]. Expanding the knowledge base on productivity impacts is a fruitful avenue for future empirical research, particularly in Africa. Comparable work has recently been done for labour productivity impacts of climate change [64].

For each sector, we calibrated a linear exposure-response functions and used these functions to translate changes in the concentration of fine particulate matter relative to the baseline into relative impacts on productivity. Based on the available literature, we derive low, medium and high sensitivity cases (see table below). Considering conventional estimates of labour supply impacts based on Ostro [65] as an alternative to productivity impacts would broaden the uncertainty range towards the lower end. In a final step, we fed these productivity changes into JRC-GEM-E3, an economy-wide, sector-specific model, to estimate the economic impacts of changes in labour productivity. The use of an economy-wide model ensures that all economic interactions are accounted for, including those related to intermediate supply chains and international trade. In the scenarios, we adjust region- and sector-specific labour productivity in the model in line with the corresponding changes in air pollution.

SI Table 10. Labour productivity impacts from selected studies expressed in percent per 10 μgm^3 change in $PM_{2.5}$ concentration. Shading indicates the values used in low, medium and high sensitivity cases. This estimate represents the impact of a 10 ppb change in ground-level ozone, and is applied accordingly in the simulations. For comparison, the change in ozone mixing ratio (pbb) is around 1-2 times the change in $PM_{2.5}$ concentration on the global level in our scenarios, but this ratio may differ substantially across countries.

First author:	Chang	Fu	He	Adhvaryu	Heyes	Kahn	Graff Zivin	Fan	Dechezleprêtre
Publication year	2016	2017	2019	2019	2016	2019	2012	2019	2019
Country of study:	US	China	China	India	US	China	US	China	EU
Industry	6.0	8.2	1.0	0.5					9.3
Services					17.1	3.5			7.1
Agriculture							5.5*	12.2	46.2

* This estimate represents the impact of a 10 ppb change in ground-level ozone, and is applied accordingly in the simulations. For comparison, the change in ozone mixing ratio (pbb) is around 1-2 times the change in PM2.5 concentration on the global level in our scenarios, but this ratio may differ substantially across countries.

The JRC-GEM-E3 model

The JRC-GEM-E3 model [66] is a computable general equilibrium model with global and economy-wide coverage, representing economic behaviour of firms, households and governments through a set of mathematical equations calibrated on historic input-output tables in the GTAP10 database [67]. It was recently applied to estimate the air quality co-benefits of climate policy through crop yield and sickness-related labour supply effects [68], which is relevant to its application here. The JRC-GEM-E3 model represents 36 countries explicitly and aggregates the rest into 6 regional groups (R) (SI Table 11).

SI Table 11. Overview of the regional aggregation of JRC-GEM-E3.

Countries			Regions
Austria	United Kingdom	Slovakia	Oceania
Belgium	Greece	Slovenia	Middle East
Bulgaria	Hungary	Sweden	Africa
Cyprus	Ireland	Romania	Other Americas
Croatia	Italy	USA	Other Asia
Czech Republic	Lithuania	Japan	Rest of Europe
Germany	Luxembourg	Canada	
Denmark	Latvia	Russian federation	
Spain	Malta	Brazil	
Estonia	Netherlands	China	
Finland	Poland	India	
France	Portugal	Korea	

To disaggregate economic impacts (ΔGDP) in a given year (t) to the country (i) level, we used the following equation:

$$\Delta GDP_{i,t} = \Delta GDP_{R,t} * \frac{\sum_s \Delta PM_{i,t} * \frac{\Delta LP_s}{\Delta PM_{i,t}} * VA^L_{s,i}}{\sum_{i\ in\ R} \sum_s \Delta PM_{i,t} * \frac{\Delta LP_s}{\Delta PM_{i,t}} * VA^L_{s,i}}$$

In this equation, $\Delta PM_{i,t}$ represents the changes in fine particulate matter concentration; $\Delta LP_s/\Delta PM_{i,t}$ is the change in labour productivity for sector s per unit of $PM_{2.5}$ concentration; $VA^L_{s,i}$ is the value added from labour in sector s in country i based on the GTAP10 database [67]. As such, the downscaling approach takes into account geographical differences in $PM_{2.5}$ changes, sector composition and labour intensity.

6. Supplementary results

SI Table 12. Global food intake by food group and diet scenario (in grams per person per day, g/d) and total energy intake by diet scenario (in kilocalories per person per day, kcal/d).

Food group	Diet scenario			
	BMK	FLX	VEG	VGN
wheat	136	108	101	101
rice	128	92	85	81
maize	44	35	33	32
grains	32	29	27	26
roots	55	51	51	51
fruits	108	200	250	300
vegetables	199	356	479	520
legumes	21	75	100	125
nuts & seeds	1	25	25	25
oils	30	26	26	26
sugar	41	25	25	25
beef	19	5	0	0
lamb	5	2	0	0
pork	30	5	0	0
poultry	29	19	0	0
eggs	20	10	10	0
milk	128	156	156	0
dairy	77	0	0	0
fish	35	20	0	0
other	7	0	0	0
total energy	2,257	2,109	2,108	2,107

SI Figure 3. Food intake (kilocalories per person per day, kcal/d) by region, diet scenario, and food group.

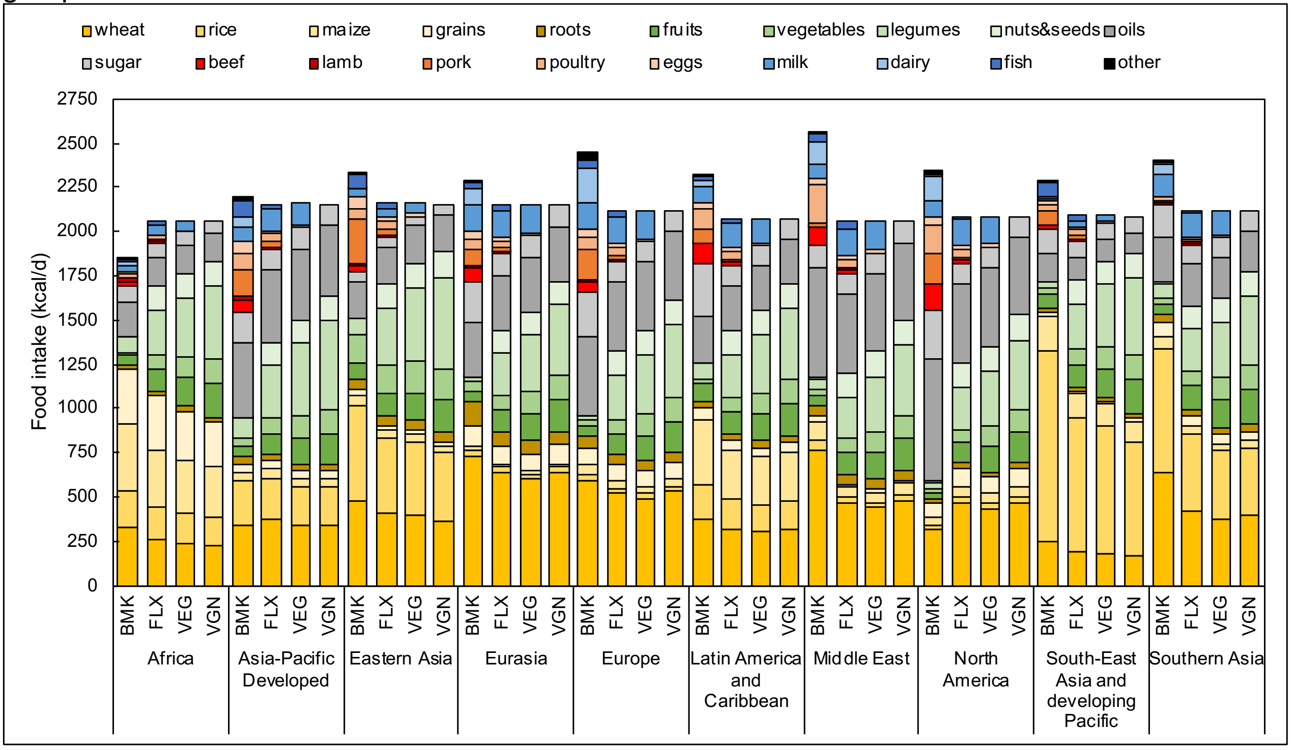

SI Table 13. Food production (kilo tonnes per year, kt/yr) by food group and diet scenario.

Food group	Diet scenario			
	BMK	FLX	VEG	VGN
wheat	924,551	759,321	711,545	681,328
rice	722,861	522,661	482,085	443,958
maize	1,199,038	842,288	727,663	503,124
grains	396,516	316,771	277,757	211,345
roots	327,838	291,958	289,476	276,908
fruits	833,672	1,661,737	2,072,807	2,481,164
vegetables	1,474,589	2,826,571	3,860,847	4,196,065
legumes / nuts	312,501	676,089	838,867	998,227
oils	626,214	567,258	565,910	561,630
sugar	287,535	208,781	208,773	208,735
beef	107,353	28,203	344	344
lamb	24,153	10,112	188	188
pork	163,435	28,015	1,083	1,083
poultry	160,312	100,641	3,126	3,126
eggs	103,164	52,707	52,707	1,420
milk	631,103	780,076	780,076	111,872
other	402,889	0	0	0

SI Figure 4. Food production (kilo tonnes per year, kt/yr) by region, diet scenario, and food group.

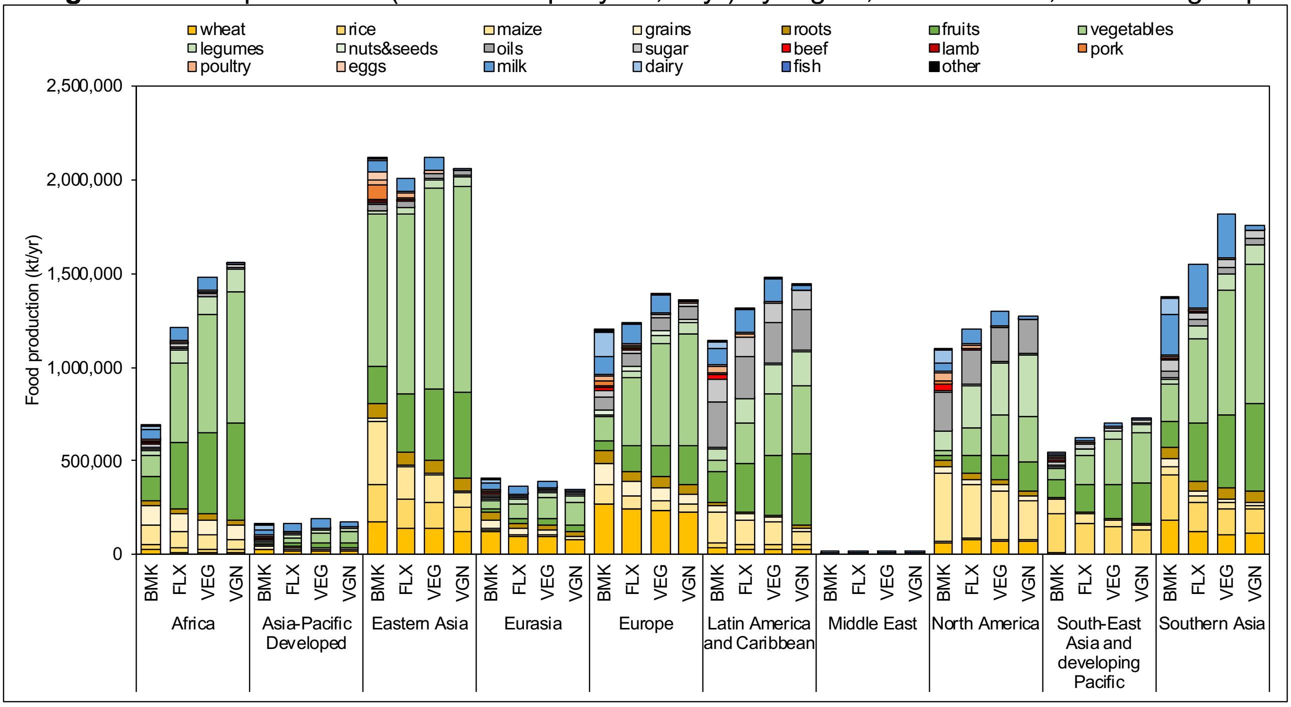

SI Table 14. Ammonia and methane emissions (Gg/yr) by food group and diet scenario in 2030.

Food group	Ammonia (Gg/yr)				Methane (Gg/yr)			
	BMK	FLX	VEG	VGN	BMK	FLX	VEG	VGN
total	49,970	27,997	14,849	7,932	159,702	83,598	49,990	23,084
staple crops	6,427	4,605	4,152	3,522	32,024	23,420	21,562	19,466
fruits&veg	460	872	1,150	1,289	0	0	0	0
legumes&nuts	425	798	963	1,124	0	0	0	0
oil&sugar	1,139	998	994	983	0	0	0	0
beef&lamb	20,404	7,533	106	106	84,286	29,972	318	318
pork&poultry	11,546	5,851	145	145	8,081	2,145	48	48
milk&eggs	9,562	7,339	7,339	763	35,311	28,062	28,062	3,252
other	8	1	1	1	0	0	0	0

SI Figure 5. Ammonia (top) and methane (bottom) emissions (Gg/yr) by food group, diet scenario, and region in 2030.

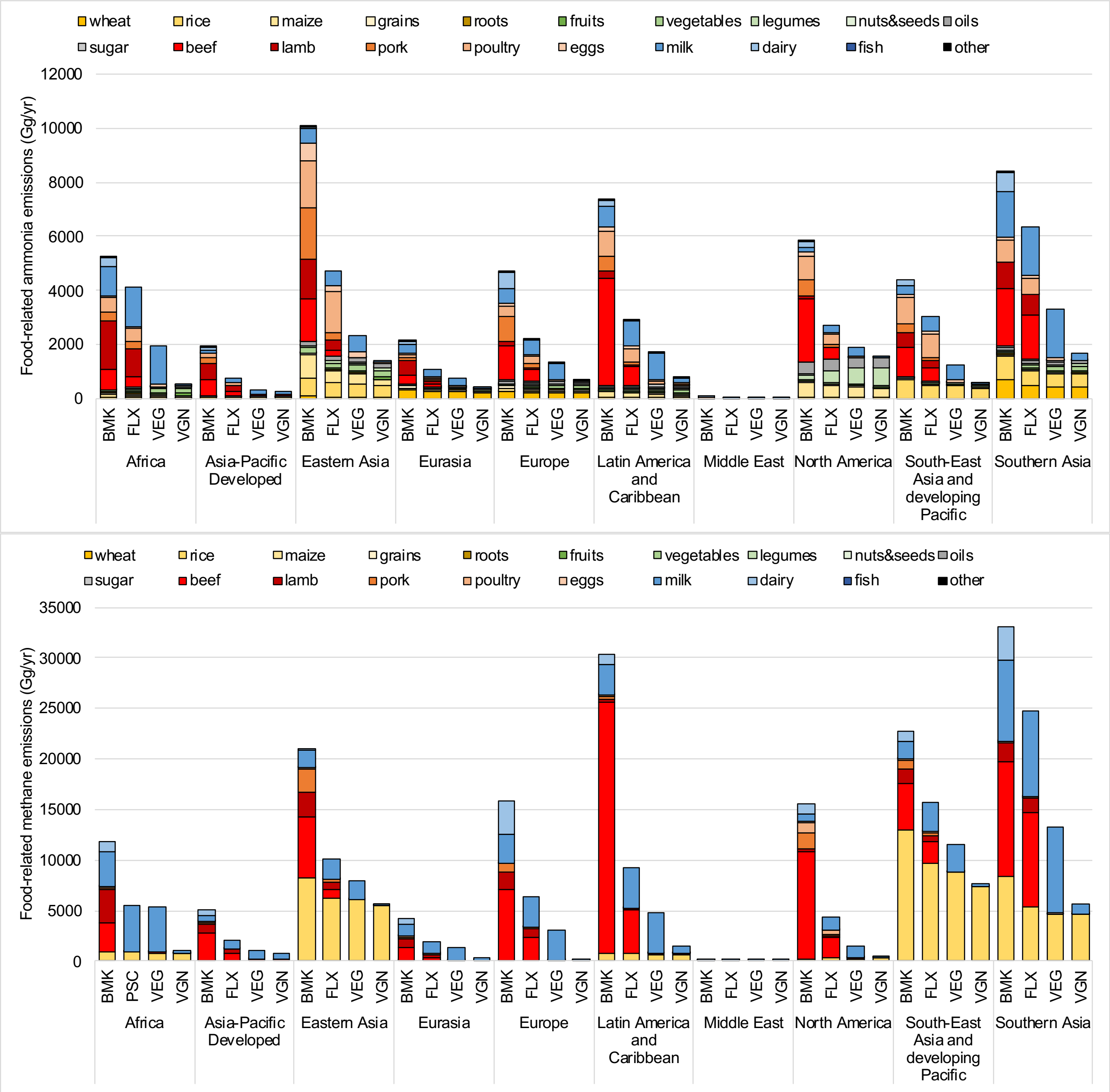

SI Figure 6. Percentage changes in particulate matter concentration (*left panels*), mortality from air pollution (*middle panels*), and economic value (*right panels*) for adoption of flexitarian (*top panels*), vegetarian (*middle panels*), and vegan (*bottom panels*) diets in 2030 compared to the baseline in 2030. Please note that the economic impacts were truncated at 1.5% of GDP. The maps were produced using the "maps" package in R (https://cran.r-project.org/web/packages/maps/index.html) and Natural Earth data for geographical outlines and borders (https://www.naturalearthdata.com/about/terms-of-use/).

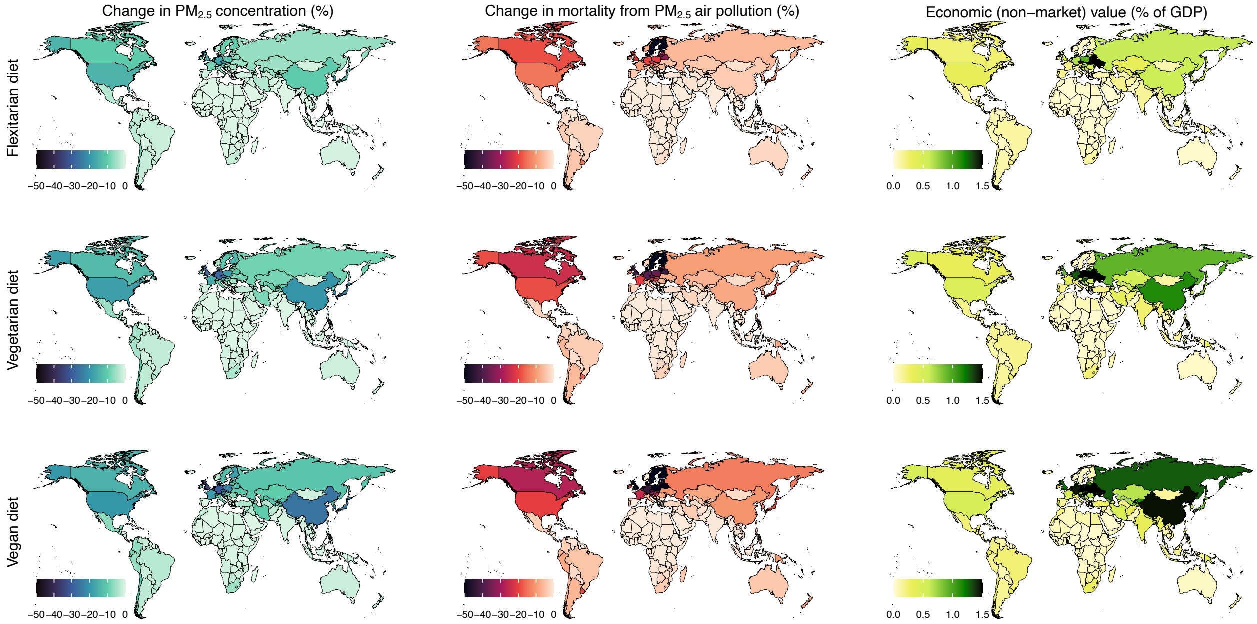

SI Figure 7. Grid-level changes in PM2.5 concentration (μgm^{-3}) in diet scenarios compared to the baseline of the same year. The maps were produced using the “maps” package in R (https://cran.r-project.org/web/packages/maps/index.html) and Natural Earth data for geographical outlines and borders (https://www.naturalearthdata.com/about/terms-of-use/).

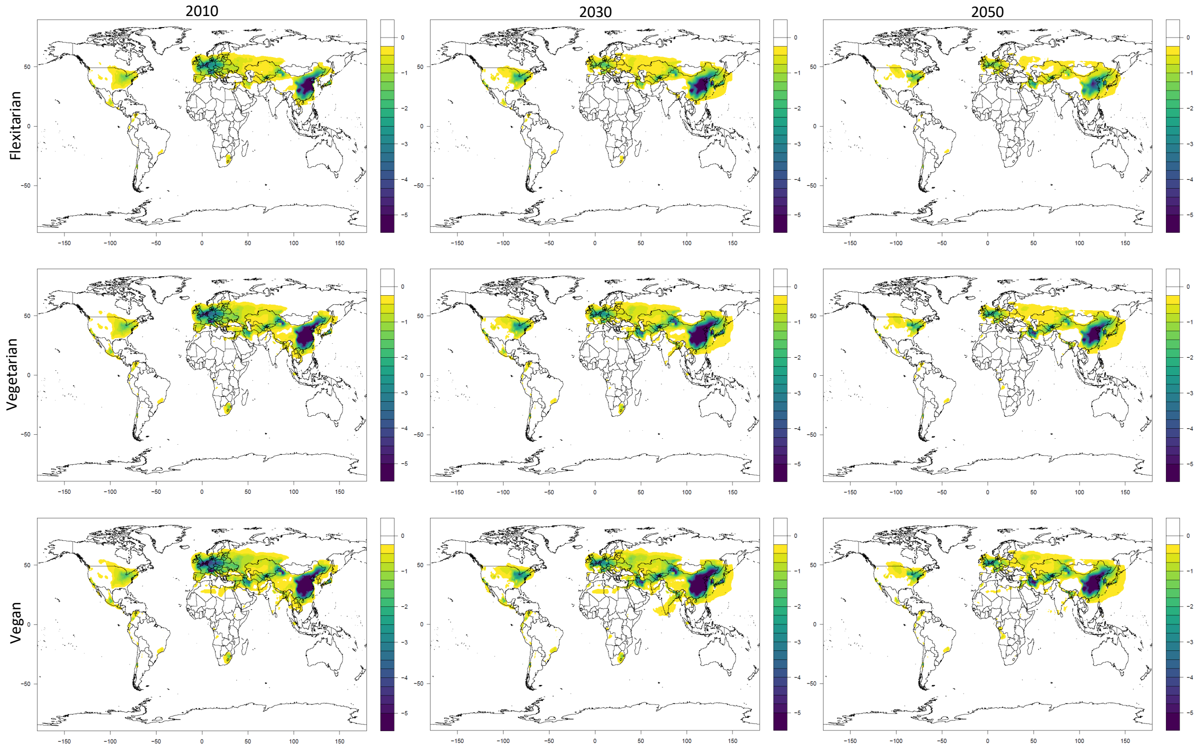

SI Table 15. Change in particulate matter concentration (PM2.5) by region and diet scenario in 2030.

Region	Change in PM2.5 (μg/m³)			Percentage change		
	FLX	VEG	VGN	FLX	VEG	VGN
Global	-1.0	-1.8	-2.4	-3%	-6%	-7%
Africa	0.0	0.0	-0.1	0%	0%	0%
Asia-Pacific Developed	-1.3	-2.0	-2.1	-12%	-20%	-21%
Eastern Asia	-4.3	-8.2	-10.3	-11%	-20%	-25%
Eurasia	-0.7	-1.1	-1.4	-5%	-8%	-10%
Europe	-0.6	-1.1	-1.3	-8%	-14%	-16%
Latin America and Caribbean	-0.3	-0.4	-0.5	-2%	-4%	-5%
Middle East	-0.4	-0.9	-1.2	-1%	-3%	-3%
North America	-1.2	-1.5	-1.6	-14%	-18%	-20%
South-East Asia and dev Pacific	0.0	-0.1	-0.1	0%	-1%	-1%
Southern Asia	0.0	-0.2	-0.4	0%	0%	-1%

SI Table 16. Change in ozone exposure by region and diet scenario in 2030.

Region	Change in ozone exposure (ppb)			Percentage change		
	FLX	VEG	VGN	FLX	VEG	VGN
Global	-1.3	-1.8	-2.3	-2%	-3%	-4%
Africa	-1.3	-1.9	-2.4	-2%	-3%	-4%
Asia-Pacific Developed	-1.1	-1.6	-2.0	-2%	-3%	-4%
Eastern Asia	-1.1	-1.5	-1.9	-2%	-2%	-3%
Eurasia	-1.2	-1.8	-2.2	-3%	-4%	-5%
Europe	-1.7	-2.4	-3.0	-3%	-5%	-6%
Latin America and Caribbean	-1.2	-1.8	-2.2	-3%	-4%	-5%
Middle East	-2.1	-3.0	-3.8	-3%	-4%	-5%
North America	-1.5	-2.1	-2.7	-3%	-4%	-5%
South-East Asia and dev Pacific	-1.1	-1.6	-2.0	-2%	-3%	-4%
Southern Asia	-1.1	-1.7	-2.1	-1%	-2%	-3%

SI Table 17. Change in premature mortality (deaths) by region, air pollutant, and diet scenario in 2030.

Region	Change in premature mortality			Percentage change			Proportion due to PM2.5		
	FLX	VEG	VGN	FLX	VEG	VGN	FLX	VEG	VGN
Global	-107,639	-188,138	-235,846	-3%	-5%	-6%	80%	83%	83%
Africa	-1,080	-2,126	-2,871	0%	-1%	-1%	7%	32%	37%
Asia-Pacific Developed	-5,337	-8,808	-9,537	-10%	-17%	-18%	94%	95%	94%
Eastern Asia	-49,229	-94,475	-120,889	-4%	-8%	-10%	86%	89%	89%
Eurasia	-5,100	-7,607	-10,279	-5%	-8%	-11%	93%	93%	94%
Europe	-19,689	-35,388	-44,064	-9%	-17%	-21%	87%	89%	89%
Latin America and Caribbean	-3,752	-5,427	-6,797	-3%	-4%	-5%	70%	70%	71%
Middle East	-822	-1,593	-2,036	-1%	-2%	-2%	60%	70%	71%
North America	-14,403	-18,997	-21,020	-12%	-16%	-18%	90%	89%	88%
South-East Asia and dev Pacific	-1,709	-3,103	-3,885	-1%	-2%	-2%	34%	48%	48%
Southern Asia	-6,516	-10,613	-14,468	0%	-1%	-1%	4%	15%	22%

SI Table 18. Global change in value (% of GDP) by diet scenario and valuation method in 2030.

Statistics	Labour productivity (% of GDP)			Value of statistical life (% of GDP)		
	FLX	VEG	VGN	FLX	VEG	VGN
Mean	0.52	0.88	1.07	0.34	0.57	0.70
Low	0.20	0.33	0.40	0.13	0.21	0.26
High	1.20	2.06	2.51	0.77	1.33	1.67

SI Figure 8. Market value of air quality changes (% of GDP) by region and diet scenario in 2030 (see SI Table 22 for values).

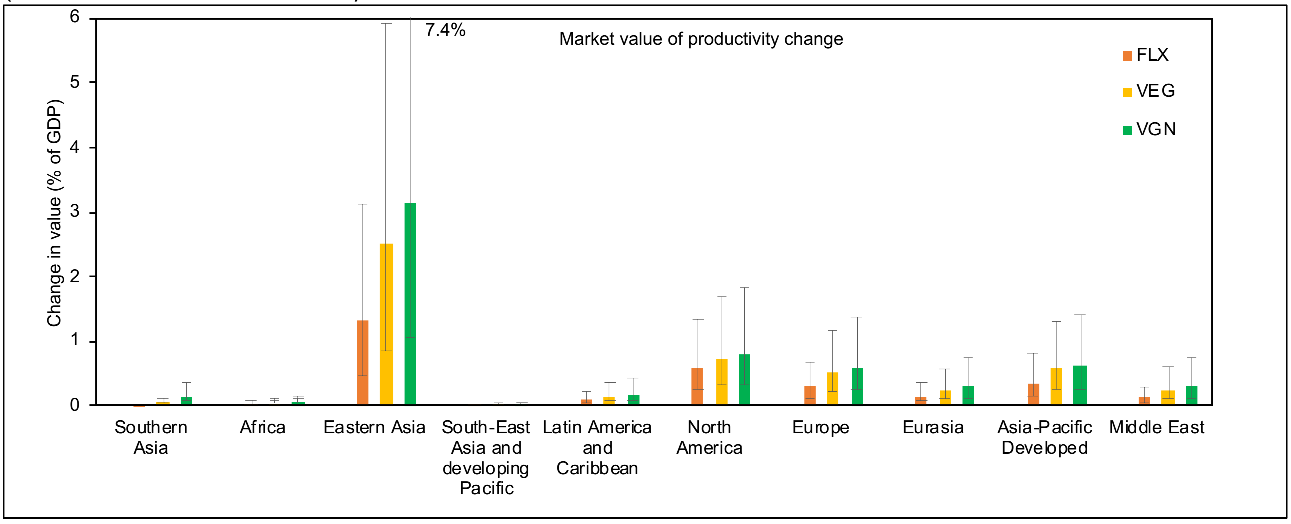

SI Figure 9. Non-market value of air quality changes (% of GDP) by region and diet scenario in 2030 (see SI Table 22 for values).

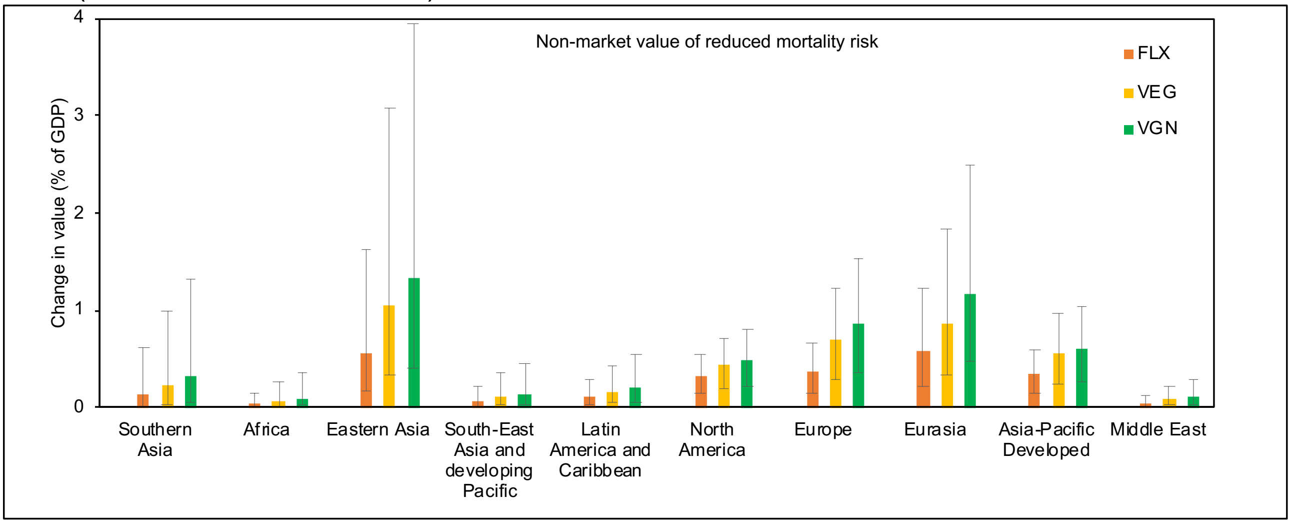

SI Table 19. Change in particulate matter concentration (PM2.5) by region and diet scenario in 2010, 2030, and 2050.

2010	Change in PM2.5 (μg/m³)			Percentage change		
Region	FLX	VEG	VGN	FLX	VEG	VGN
Global	-1.3	-2.1	-2.3	-4%	-7%	-7%
Africa	0.0	-0.1	-0.1	0%	0%	-1%
Asia-Pacific Developed	-1.0	-1.3	-1.4	-9%	-12%	-13%
Eastern Asia	-4.6	-7.7	-8.2	-10%	-16%	-17%
Eurasia	-0.6	-0.9	-1.2	-4%	-6%	-8%
Europe	-1.6	-2.0	-2.4	-13%	-17%	-20%
Latin America and Caribbean	-0.3	-0.4	-0.4	-2%	-3%	-4%
Middle East	-0.4	-0.8	-1.1	-1%	-2%	-3%
North America	-0.7	-0.8	-0.9	-8%	-9%	-9%
South-East Asia and developing Pacific	-0.1	-0.2	-0.3	-1%	-2%	-2%
Southern Asia	0.0	-0.2	-0.3	0%	0%	-1%

2030	Change in PM2.5 (μg/m³)			Percentage change		
Region	FLX	VEG	VGN	FLX	VEG	VGN
Global	-1.0	-1.8	-2.4	-3%	-6%	-7%
Africa	0.0	0.0	-0.1	0%	0%	0%
Asia-Pacific Developed	-1.3	-2.0	-2.1	-12%	-20%	-21%
Eastern Asia	-4.3	-8.2	-10.3	-11%	-20%	-25%
Eurasia	-0.7	-1.1	-1.4	-5%	-8%	-10%
Europe	-0.6	-1.1	-1.3	-8%	-14%	-16%
Latin America and Caribbean	-0.3	-0.4	-0.5	-2%	-4%	-5%
Middle East	-0.4	-0.9	-1.2	-1%	-3%	-3%
North America	-1.2	-1.5	-1.6	-14%	-18%	-20%
South-East Asia and developing Pacific	0.0	-0.1	-0.1	0%	-1%	-1%
Southern Asia	0.0	-0.2	-0.4	0%	0%	-1%

2050	Change in PM2.5 (μg/m³)			Percentage change		
Region	FLX	VEG	VGN	FLX	VEG	VGN
Global	-0.4	-0.8	-1.2	-2%	-3%	-4%
Africa	0.1	0.0	0.0	0%	0%	0%
Asia-Pacific Developed	-0.4	-0.7	-0.8	-5%	-9%	-11%
Eastern Asia	-2.2	-4.7	-6.5	-7%	-15%	-21%
Eurasia	-0.5	-0.8	-1.1	-4%	-7%	-9%
Europe	-0.5	-0.8	-0.9	-7%	-11%	-13%
Latin America and Caribbean	-0.2	-0.2	-0.3	-2%	-3%	-4%
Middle East	-0.5	-0.9	-1.2	-1%	-3%	-3%
North America	-0.9	-1.1	-1.2	-13%	-16%	-17%
South-East Asia and developing Pacific	0.0	0.0	0.0	0%	0%	0%
Southern Asia	0.2	0.3	0.3	0%	1%	1%

SI Table 20. Change in ozone exposure by region and diet scenario in 2010, 2030, and 2050.

2010	Change in O_3 exposure (ppb)			Percentage change		
Region	FLX	VEG	VGN	FLX	VEG	VGN
Global	-1.0	-1.4	-1.8	-2%	-2%	-3%
Africa	-1.1	-1.6	-1.9	-2%	-3%	-3%
Asia-Pacific Developed	-0.9	-1.3	-1.5	-2%	-3%	-3%
Eastern Asia	-0.8	-1.2	-1.5	-1%	-2%	-2%
Eurasia	-1.0	-1.4	-1.7	-2%	-3%	-4%
Europe	-1.3	-1.9	-2.3	-2%	-4%	-4%
Latin America and Caribbean	-1.0	-1.4	-1.7	-2%	-3%	-4%
Middle East	-1.6	-2.4	-2.9	-2%	-4%	-4%
North America	-1.2	-1.7	-2.1	-2%	-3%	-4%
South-East Asia and developing Pacific	-0.9	-1.3	-1.5	-2%	-3%	-3%
Southern Asia	-0.9	-1.3	-1.6	-1%	-2%	-2%

2030	Change in O_3 exposure (ppb)			Percentage change		
Region	FLX	VEG	VGN	FLX	VEG	VGN
Global	-1.3	-1.8	-2.3	-2%	-3%	-4%
Africa	-1.3	-1.9	-2.4	-2%	-3%	-4%
Asia-Pacific Developed	-1.1	-1.6	-2.0	-2%	-3%	-4%
Eastern Asia	-1.1	-1.5	-1.9	-2%	-2%	-3%
Eurasia	-1.2	-1.8	-2.2	-3%	-4%	-5%
Europe	-1.7	-2.4	-3.0	-3%	-5%	-6%
Latin America and Caribbean	-1.2	-1.8	-2.2	-3%	-4%	-5%
Middle East	-2.1	-3.0	-3.8	-3%	-4%	-5%
North America	-1.5	-2.1	-2.7	-3%	-4%	-5%
South-East Asia and developing Pacific	-1.1	-1.6	-2.0	-2%	-3%	-4%
Southern Asia	-1.1	-1.7	-2.1	-1%	-2%	-3%

2050	Change in O_3 exposure (ppb)			Percentage change		
Region	FLX	VEG	VGN	FLX	VEG	VGN
Global	-1.0	-1.5	-1.9	-2%	-3%	-3%
Africa	-1.0	-1.6	-2.0	-2%	-3%	-4%
Asia-Pacific Developed	-0.9	-1.3	-1.6	-2%	-3%	-4%
Eastern Asia	-0.8	-1.3	-1.6	-2%	-2%	-3%
Eurasia	-1.0	-1.5	-1.9	-2%	-3%	-4%
Europe	-1.3	-2.0	-2.5	-3%	-4%	-5%
Latin America and Caribbean	-1.0	-1.5	-1.9	-2%	-3%	-4%
Middle East	-1.7	-2.5	-3.2	-3%	-4%	-5%
North America	-1.2	-1.8	-2.3	-2%	-3%	-4%
South-East Asia and developing Pacific	-0.9	-1.3	-1.7	-2%	-3%	-4%
Southern Asia	-0.9	-1.4	-1.8	-1%	-2%	-2%

SI Table 21. Change in premature mortality (deaths) by region, air pollutant, and diet scenario in 2010, 2030, and 2050.

2010	Change in premature mortality			Percentage change		
Region	FLX	VEG	VGN	FLX	VEG	VGN
Global	-78,556	-119,701	-138,121	-2%	-4%	-4%
Africa	-991	-1,854	-2,292	0%	-1%	-1%
Asia-Pacific Developed	-2,829	-4,023	-4,210	-7%	-9%	-10%
Eastern Asia	-32,561	-55,000	-59,968	-3%	-5%	-6%
Eurasia	-4,355	-6,267	-7,940	-3%	-5%	-6%
Europe	-23,571	-31,653	-39,168	-9%	-12%	-15%
Latin America and Caribbean	-2,394	-3,355	-3,975	-2%	-3%	-3%
Middle East	-370	-740	-966	-1%	-1%	-2%
North America	-6,258	-7,497	-7,908	-5%	-6%	-7%
South-East Asia and developing Pacific	-1,372	-2,655	-3,106	-1%	-2%	-2%
Southern Asia	-3,854	-6,657	-8,590	0%	-1%	-1%

2030	Change in premature mortality			Percentage change		
Region	FLX	VEG	VGN	FLX	VEG	VGN
Global	-107,639	-188,138	-235,846	-3%	-5%	-6%
Africa	-1,080	-2,126	-2,871	0%	-1%	-1%
Asia-Pacific Developed	-5,337	-8,808	-9,537	-10%	-17%	-18%
Eastern Asia	-49,229	-94,475	-120,889	-4%	-8%	-10%
Eurasia	-5,100	-7,607	-10,279	-5%	-8%	-11%
Europe	-19,689	-35,388	-44,064	-9%	-17%	-21%
Latin America and Caribbean	-3,752	-5,427	-6,797	-3%	-4%	-5%
Middle East	-822	-1,593	-2,036	-1%	-2%	-2%
North America	-14,403	-18,997	-21,020	-12%	-16%	-18%
South-East Asia and developing Pacific	-1,709	-3,103	-3,885	-1%	-2%	-2%
Southern Asia	-6,516	-10,613	-14,468	0%	-1%	-1%

2050	Change in premature mortality			Percentage change		
Region	FLX	VEG	VGN	FLX	VEG	VGN
Global	-100,249	-170,724	-222,175	-2%	-4%	-5%
Africa	-169	-1,245	-2,143	0%	0%	0%
Asia-Pacific Developed	-2,492	-4,217	-4,874	-6%	-10%	-11%
Eastern Asia	-38,802	-78,461	-108,252	-3%	-6%	-9%
Eurasia	-4,761	-7,317	-10,703	-5%	-8%	-11%
Europe	-24,810	-37,863	-45,347	-13%	-20%	-24%
Latin America and Caribbean	-4,015	-5,862	-7,638	-2%	-4%	-5%
Middle East	-1,519	-2,777	-3,544	-1%	-2%	-2%
North America	-16,754	-21,192	-23,932	-15%	-19%	-21%
South-East Asia and developing Pacific	-1,288	-2,453	-3,224	-1%	-1%	-2%
Southern Asia	-5,638	-9,337	-12,517	0%	0%	-1%

SI Table 22. Change in market and non-market value (% of GDP) by diet scenario in 2030 and 2050.

2030	Non-market value (% of GDP)			GDP (market) impact (%)		
Region	FLX	VEG	VGN	FLX	VEG	VGN
Global	0.34	0.57	0.70	0.52	0.88	1.07
Africa	0.04	0.07	0.10	0.01	0.03	0.05
Asia-Pacific Developed	0.34	0.56	0.61	0.36	0.57	0.61
Eastern Asia	0.55	1.05	1.33	1.33	2.51	3.16
Eurasia	0.58	0.86	1.17	0.15	0.23	0.30
Europe	0.38	0.69	0.86	0.30	0.51	0.60
Latin America and Caribbean	0.11	0.16	0.20	0.09	0.15	0.18
Middle East	0.05	0.09	0.11	0.12	0.26	0.32
North America	0.33	0.44	0.48	0.58	0.74	0.79
South-East Asia and developing Pacific	0.06	0.12	0.15	0.01	0.02	0.03
Southern Asia	0.14	0.23	0.32	0.00	0.05	0.14

2050	Non-market value (% of GDP)			GDP (market) impact (%)		
Region	FLX	VEG	VGN	FLX	VEG	VGN
Global	0.28	0.46	0.58	0.29	0.49	0.63
Africa	-0.01	0.01	0.04	-0.02	-0.01	0.00
Asia-Pacific Developed	0.15	0.25	0.29	0.12	0.20	0.23
Eastern Asia	0.44	0.87	1.20	0.69	1.42	1.97
Eurasia	0.47	0.72	1.05	0.08	0.11	0.14
Europe	0.44	0.68	0.81	0.24	0.36	0.43
Latin America and Caribbean	0.10	0.15	0.20	0.05	0.08	0.11
Middle East	0.07	0.12	0.16	0.13	0.24	0.30
North America	0.33	0.41	0.47	0.46	0.56	0.61
South-East Asia and developing Pacific	0.04	0.07	0.09	0.00	0.00	0.00
Southern Asia	0.09	0.16	0.21	-0.06	-0.10	-0.10

SI Table 23. Premature mortality (deaths) from air pollution by region and diet scenario in 2010 for a sensitivity analysis in which NH_3 emissions were decreased/increased by 20%.

2010		Change in premature mortality			Percentage change		
Region	NH_3 emissions	FLX	VEG	VGN	FLX	VEG	VGN
Global	-20%	-71,253	-103,859	-118,761	-2%	-3%	-4%
	main	-78,540	-119,681	-138,118	-2%	-4%	-4%
	+20%	-83,814	-132,085	-154,370	-3%	-4%	-5%
Africa	-20%	-941	-1,724	-2,111	0%	-1%	-1%
	main	-991	-1,854	-2,291	0%	-1%	-1%
	+20%	-1,011	-1,944	-2,411	0%	-1%	-1%
Asia-Pacific Developed	-20%	-2,163	-2,946	-3,089	-5%	-7%	-7%
	main	-2,829	-4,023	-4,210	-7%	-9%	-10%
	+20%	-3,406	-5,060	-5,319	-8%	-12%	-12%
Eastern Asia	-20%	-29,602	-46,018	-49,792	-3%	-5%	-5%
	main	-32,552	-54,987	-59,962	-3%	-5%	-6%
	+20%	-34,673	-61,997	-68,611	-3%	-6%	-7%
Eurasia	-20%	-3,815	-5,437	-6,810	-3%	-4%	-5%
	main	-4,355	-6,267	-7,940	-3%	-5%	-6%
	+20%	-4,775	-6,947	-8,900	-4%	-5%	-7%
Europe	-20%	-21,731	-28,583	-34,468	-8%	-11%	-13%
	main	-23,571	-31,653	-39,168	-9%	-12%	-15%
	+20%	-24,651	-33,713	-42,818	-9%	-12%	-16%
Latin America and Caribbean	-20%	-2,175	-3,035	-3,565	-2%	-3%	-3%
	main	-2,395	-3,355	-3,975	-2%	-3%	-3%
	+20%	-2,555	-3,625	-4,315	-2%	-3%	-4%
Middle East	-20%	-336	-658	-853	-1%	-1%	-1%
	main	-370	-740	-966	-1%	-1%	-2%
	+20%	-399	-812	-1,068	-1%	-1%	-2%
North America	-20%	-5,371	-6,472	-6,864	-5%	-6%	-6%
	main	-6,258	-7,498	-7,908	-5%	-6%	-7%
	+20%	-7,055	-8,423	-8,850	-6%	-7%	-7%
South-East Asia and dev Pacific	-20%	-1,293	-2,446	-2,826	-1%	-1%	-2%
	main	-1,373	-2,656	-3,106	-1%	-2%	-2%
	+20%	-1,413	-2,826	-3,316	-1%	-2%	-2%
Southern Asia	-20%	-3,827	-6,539	-8,383	0%	-1%	-1%
	main	-3,847	-6,649	-8,593	0%	-1%	-1%
	+20%	-3,877	-6,739	-8,763	0%	-1%	-1%

SI Table 24. Premature mortality (deaths) from air pollution by region and diet scenario in 2030 for a sensitivity analysis in which NH_3 emissions were decreased/increased by 20%.

2030		Change in premature mortality			Percentage change		
Region	NH_3 emissions	FLX	VEG	VGN	FLX	VEG	VGN
Global	-20%	-105,698	-178,239	-216,591	-3%	-4%	-5%
	main	-107,641	-188,137	-235,840	-3%	-5%	-6%
	+20%	-107,275	-193,394	-248,884	-3%	-5%	-6%
Africa	-20%	-1,141	-2,136	-2,821	0%	-1%	-1%
	main	-1,081	-2,126	-2,871	0%	-1%	-1%
	+20%	-991	-2,056	-2,851	0%	-1%	-1%
Asia-Pacific Developed	-20%	-5,024	-7,523	-8,082	-10%	-15%	-16%
	main	-5,337	-8,808	-9,537	-10%	-17%	-18%
	+20%	-5,166	-9,585	-10,501	-10%	-18%	-20%
Eastern Asia	-20%	-48,117	-89,457	-110,338	-4%	-7%	-9%
	main	-49,238	-94,479	-120,889	-4%	-8%	-10%
	+20%	-49,179	-97,181	-128,010	-4%	-8%	-10%
Eurasia	-20%	-4,879	-7,062	-9,293	-5%	-7%	-10%
	main	-5,100	-7,607	-10,279	-5%	-8%	-11%
	+20%	-5,135	-7,913	-10,977	-5%	-8%	-11%
Europe	-20%	-21,789	-35,979	-42,275	-11%	-17%	-20%
	main	-19,689	-35,389	-44,064	-9%	-17%	-21%
	+20%	-17,269	-33,578	-44,314	-8%	-16%	-21%
Latin America and Caribbean	-20%	-3,552	-5,037	-6,238	-2%	-3%	-4%
	main	-3,752	-5,427	-6,798	-3%	-4%	-5%
	+20%	-3,882	-5,697	-7,228	-3%	-4%	-5%
Middle East	-20%	-765	-1,444	-1,830	-1%	-2%	-2%
	main	-822	-1,593	-2,036	-1%	-2%	-2%
	+20%	-867	-1,718	-2,213	-1%	-2%	-3%
North America	-20%	-12,252	-16,039	-17,679	-11%	-14%	-16%
	main	-14,403	-18,997	-21,020	-12%	-16%	-18%
	+20%	-16,546	-21,845	-24,174	-14%	-18%	-20%
South-East Asia and dev Pacific	-20%	-1,669	-2,953	-3,675	-1%	-2%	-2%
	main	-1,709	-3,103	-3,885	-1%	-2%	-2%
	+20%	-1,729	-3,213	-4,055	-1%	-2%	-2%
Southern Asia	-20%	-6,510	-10,609	-14,360	0%	-1%	-1%
	main	-6,510	-10,609	-14,460	0%	-1%	-1%
	+20%	-6,510	-10,609	-14,560	0%	-1%	-1%

SI Table 25. Premature mortality (deaths) from air pollution by region and diet scenario in 2050 for a sensitivity analysis in which NH_3 emissions were decreased/increased by 20%.

2050		Change in premature mortality			Percentage change		
Region	NH_3 emissions	FLX	VEG	VGN	FLX	VEG	VGN
Global	-20%	-101,015	-165,825	-205,703	-2%	-4%	-4%
	main	-100,251	-170,727	-222,184	-2%	-4%	-5%
	+20%	-96,110	-170,230	-231,298	-2%	-4%	-5%
Africa	-20%	-469	-1,585	-2,433	0%	0%	0%
	main	-169	-1,245	-2,143	0%	0%	0%
	+20%	161	-845	-1,783	0%	0%	0%
Asia-Pacific Developed	-20%	-2,579	-3,780	-4,295	-6%	-9%	-10%
	main	-2,492	-4,217	-4,874	-6%	-10%	-11%
	+20%	-2,253	-4,412	-5,212	-5%	-10%	-12%
Eastern Asia	-20%	-40,843	-78,460	-100,566	-3%	-7%	-8%
	main	-38,803	-78,460	-108,256	-3%	-6%	-9%
	+20%	-35,733	-76,270	-112,396	-3%	-6%	-9%
Eurasia	-20%	-4,664	-6,979	-9,733	-5%	-7%	-10%
	main	-4,761	-7,317	-10,703	-5%	-8%	-11%
	+20%	-4,677	-7,295	-11,143	-5%	-7%	-11%
Europe	-20%	-25,930	-36,674	-41,648	-14%	-20%	-22%
	main	-24,810	-37,863	-45,347	-13%	-20%	-24%
	+20%	-22,290	-37,143	-46,746	-12%	-19%	-24%
Latin America and Caribbean	-20%	-3,865	-5,562	-7,108	-2%	-3%	-4%
	main	-4,015	-5,862	-7,638	-2%	-4%	-5%
	+20%	-4,085	-6,052	-8,018	-2%	-4%	-5%
Middle East	-20%	-1,449	-2,567	-3,235	-1%	-2%	-2%
	main	-1,519	-2,777	-3,545	-1%	-2%	-2%
	+20%	-1,539	-2,907	-3,765	-1%	-2%	-3%
North America	-20%	-14,259	-18,143	-20,609	-13%	-17%	-19%
	main	-16,754	-21,192	-23,932	-15%	-19%	-21%
	+20%	-18,777	-23,691	-26,739	-16%	-20%	-23%
South-East Asia and dev Pacific	-20%	-1,318	-2,433	-3,155	-1%	-1%	-2%
	main	-1,288	-2,453	-3,225	-1%	-1%	-2%
	+20%	-1,278	-2,473	-3,275	-1%	-1%	-2%
Southern Asia	-20%	-5,638	-9,642	-12,921	0%	0%	-1%
	main	-5,638	-9,342	-12,521	0%	0%	-1%
	+20%	-5,638	-9,142	-12,221	0%	0%	-1%

SI Table 26. Percentage changes in consumption and production for dietary changes to flexitarian diets in 2030 for selected countries and regions.

Food group	Europe		USA		China	
	consumption	production	consumption	production	consumption	production
wheat	-13%	-11%	47%	39%	-16%	-20%
rice	-15%	-17%	38%	14%	-22%	-24%
maize	-19%	-31%	37%	-21%	-22%	-48%
grains	-1%	-31%	40%	-10%	-18%	-41%
roots	-14%	-16%	0%	0%	0%	-6%
fruits	111%	138%	235%	264%	39%	55%
vegetables	134%	178%	384%	381%	0%	18%
legumes	899%	374%	761%	115%	281%	100%
sugar	-55%	-36%	-57%	-57%	0%	0%
beef	-80%	-67%	-89%	-81%	-87%	-86%
lamb	-78%	-51%	-89%	-65%	-87%	-75%
pork	-86%	-81%	-89%	-84%	-87%	-87%
poultry	-32%	-29%	-71%	-61%	-9%	-9%
eggs	-57%	-48%	-53%	-50%	-71%	-69%
milk	-1%	6%	-33%	-29%	0%	0%

Supplementary references

1. Himics, M., Fellmann, T. & Barreiro-Hurle, J. Setting Climate Action as the Priority for the Common Agricultural Policy: A Simulation Experiment. *Journal of Agricultural Economics* **71**, 50–69 (2020).
2. Jansson, T. *et al.* Coupled Agricultural Subsidies in the EU Undermine Climate Efforts. *Applied Economic Perspectives and Policy* **43**, 1503–1519 (2021).
3. Britz, W. & Witzke, P. CAPRI model documentation 2014. 277 (2014).
4. M'barek, R. *et al. Scenar 2030 - Pathways for the European agriculture and food sector beyond 2020*. (Publication Office of the European Union, 2017). doi:10.2760/887521.
5. O'Neill, B. C. *et al.* The roads ahead: Narratives for shared socioeconomic pathways describing world futures in the 21st century. *Global Environmental Change* (2014) doi:10.1016/j.gloenvcha.2015.01.004.
6. van Vuuren, D. P. *et al.* A new scenario framework for Climate Change Research: Scenario matrix architecture. *Climatic Change* **122**, 373–386 (2014).
7. Hasegawa, T. *et al.* Risk of increased food insecurity under stringent global climate change mitigation policy. *Nature Climate Change* **8**, 699–703 (2018).
8. Frank, S. *et al.* Agricultural non-CO2 emission reduction potential in the context of the 1.5 °C target. *Nature Climate Change* **9**, 66–72 (2019).
9. Warszawski, L. *et al.* The Inter-Sectoral Impact Model Intercomparison Project (ISI–MIP): Project framework. *Proceedings of the National Academy of Sciences* **111**, 3228 LP – 3232 (2014).
10. Witzke, P. *et al.* Assessing the Importance of Technological non-CO2 GHG Emission Mitigation Options in EU Agriculture with the CAPRI model. in *EAAE 2014 Congress 'Agri-Food and Rural Innovations for Healthier Societies'. August 26 to 29, 2014 Ljubljana, Slovenia* 1–15 (2014).
11. Fellmann, T. *et al.* Major challenges of integrating agriculture into climate change mitigation policy frameworks. *Mitigation and Adaptation Strategies for Global Change* **23**, 451–468 (2018).
12. Pérez Domínguez, I. *et al. An economic assessment of GHG mitigation policy options for EU agriculture (EcAMPA 2)*. *JRC Science for Policy Report* vol. EUR27973 (2016).
13. Willett, W. *et al.* Food in the Anthropocene: the EAT–Lancet Commission on healthy diets from sustainable food systems. *The Lancet* **393**, 447–492 (2019).
14. Springmann, M. *et al.* Options for keeping the food system within environmental limits. *Nature* **562**, 519–525 (2018).
15. Pérez, D. I. *et al.* Agricultural GHG emissions in the EU: an exploratory economic assessment of mitigation policy options. *JRC Publications Repository* https://publications.jrc.ec.europa.eu/repository/handle/JRC69817 (2012) doi:10.2791/8124.
16. Leip, A. *et al.* Impacts of European livestock production: nitrogen, sulphur, phosphorus and greenhouse gas emissions, land-use, water eutrophication and biodiversity. *Environmental Research Letters* **10**, 115004 (2015).
17. Fellmann, T. *et al.* Major challenges of integrating agriculture into climate change mitigation policy frameworks. *Mitigation and Adaptation Strategies for Global Change* **23**, 451–468 (2018).
18. Jansson, T. *et al. Coupled agricultural subsidies in the EU undermine climate efforts. Agrifood Economics Centre working paper*. (AgriFood economics centre, 2018).
19. Leip, A. Quantitative quality assessment of the greenhouse gas inventory for agriculture in Europe. *Climatic Change* **103**, 245–261 (2010).
20. Leip, A., Weiss, F., Lesschen, J. P. & Westhoek, H. The nitrogen footprint of food products in the European Union. *The Journal of Agricultural Science* **152**, 20–33 (2014).

21. Ivanovich, C. C., Sun, T., Gordon, D. R. & Ocko, I. B. Future warming from global food consumption. *Nat. Clim. Chang.* **13**, 297–302 (2023).
22. Weiss, F. & Leip, A. Greenhouse gas emissions from the EU livestock sector: A life cycle assessment carried out with the CAPRI model. *Agriculture, Ecosystems & Environment* **149**, 124–134 (2012).
23. Weiss, F. *et al.* CAPRI development of Greenhouse Gas and Life Cycle Analysis module. JRC Technical Report. (2015).
24. Fang, Y., Naik, V., Horowitz, L. W. & Mauzerall, D. L. Air pollution and associated human mortality: the role of air pollutant emissions, climate change and methane concentration increases from the preindustrial period to present. *Atmospheric Chemistry and Physics* **13**, 1377–1394 (2013).
25. West, J. J., Fiore, A. M., Horowitz, L. W. & Mauzerall, D. L. Global health benefits of mitigating ozone pollution with methane emission controls. *Proceedings of the National Academy of Sciences* **103**, 3988–3993 (2006).
26. Van Dingenen, R. *et al.* TM5-FASST: A global atmospheric source-receptor model for rapid impact analysis of emission changes on air quality and short-lived climate pollutants. *Atmospheric Chemistry and Physics* **18**, 16173–16211 (2018).
27. Krol, M. *et al.* The two-way nested global chemistry-transport zoom model TM5: algorithm and applications. *Atmos. Chem. Phys.* **5**, 417–432 (2005).
28. Liu, Z. *et al.* The nonlinear response of fine particulate matter pollution to ammonia emission reductions in North China. *Environ. Res. Lett.* **16**, 034014 (2021).
29. Thunis, P. *et al.* Non-linear response of $PM_{2.5}$ to changes in NO_x and NH_3 emissions in the Po basin (Italy): consequences for air quality plans. *Atmospheric Chemistry and Physics* **21**, 9309–9327 (2021).
30. Himics, M. *et al.* Co-benefits of a flexitarian diet for air quality and human health in Europe. *Ecological Economics* **191**, 107232 (2022).
31. Rao, S. *et al.* Better air for better health: Forging synergies in policies for energy access, climate change and air pollution. *Global Environmental Change* **23**, 1122–1130 (2013).
32. Gidden, M. J. *et al.* Global emissions pathways under different socioeconomic scenarios for use in CMIP6: a dataset of harmonized emissions trajectories through the end of the century. *Geoscientific Model Development* **12**, 1443–1475 (2019).
33. Jones, B. & O'Neill, B. C. Spatially explicit global population scenarios consistent with the Shared Socioeconomic Pathways. *Environmental Research Letters* **11**, 084003 (2016).
34. Burnett, R. T. *et al.* An Integrated Risk Function for Estimating the Global Burden of Disease Attributable to Ambient Fine Particulate Matter Exposure. *Environmental Health Perspectives* (2014) doi:10.1289/ehp.1307049.
35. Cohen, A. J. *et al.* Estimates and 25-year trends of the global burden of disease attributable to ambient air pollution: an analysis of data from the Global Burden of Diseases Study 2015. *The Lancet* **389**, 1907–1918 (2017).
36. Stanaway, J. D. *et al.* Global, regional, and national comparative risk assessment of 84 behavioural, environmental and occupational, and metabolic risks or clusters of risks for 195 countries and territories, 1990–2017: a systematic analysis for the Global Burden of Disease Study 2017. *The Lancet* **392**, 1923–1994 (2018).
37. Institute for Health Metrics and Evaluation. GBD Compare | IHME Viz Hub. http://vizhub.healthdata.org/gbd-compare (2018).
38. Institute for Health Metrics and Evaluation. GBD Foresight | IHME Viz Hub. http://vizhub.healthdata.org/gbd-foresight/forecasting (2016).

39. Van Dingenen, R. *et al.* TM5-FASST: a global atmospheric source–receptor model for rapid impact analysis of emission changes on air quality and short-lived climate pollutants. *Atmospheric Chemistry and Physics* **18**, 16173–16211 (2018).
40. Punger, E. M. & West, J. J. The effect of grid resolution on estimates of the burden of ozone and fine particulate matter on premature mortality in the USA. *Air Qual Atmos Health* **6**, 563–573 (2013).
41. Reis, L. A., Drouet, L. & Tavoni, M. Internalising health-economic impacts of air pollution into climate policy: a global modelling study. *The Lancet Planetary Health* **6**, e40–e48 (2022).
42. Chang, T., Graff Zivin, J., Gross, T. & Neidell, M. Particulate Pollution and the Productivity of Pear Packers. *American Economic Journal: Economic Policy* **8**, 141–169 (2016).
43. Fu, S., Viard, B. & Zhang, P. *Air Quality and Manufacturing Firm Productivity: Comprehensive Evidence from China*. *MPRA Paper* https://ideas.repec.org/p/pra/mprapa/78914.html (2017).
44. He, J., Liu, H. & Salvo, A. Severe Air Pollution and Labor Productivity: Evidence from Industrial Towns in China. *American Economic Journal: Applied Economics* **11**, 173–201 (2019).
45. Adhvaryu, A., Kala, N. & Nyshadham, A. *Management and Shocks to Worker Productivity*. https://papers.ssrn.com/abstract=3395629 (2019).
46. Heyes, A., Neidell, M. & Saberian, S. *The Effect of Air Pollution on Investor Behavior: Evidence from the S&P 500*. *NBER Working Papers* https://ideas.repec.org/p/nbr/nberwo/22753.html (2016).
47. Kahn, M. E. & Li, P. Air pollution lowers high skill public sector worker productivity in China. *Environ. Res. Lett.* **15**, 084003 (2020).
48. Graff Zivin, J. & Neidell, M. The Impact of Pollution on Worker Productivity. *American Economic Review* **102**, 3652–3673 (2012).
49. Fan, M. & Grainger, C. The impact of air pollution on labor supply in China. *Work. Pap., Univ. Wis., Madison Google Scholar Article Location* (2019).
50. Dechezleprêtre, A., Rivers, N. & Stadler, B. *The economic cost of air pollution: Evidence from Europe*. https://www.oecd-ilibrary.org/economics/the-economic-cost-of-air-pollution-evidence-from-europe_56119490-en (2019) doi:10.1787/56119490-en.
51. Lichter, A., Pestel, N. & Sommer, E. Productivity effects of air pollution: Evidence from professional soccer. *Labour Economics* **48**, 54–66 (2017).
52. Sager, L. Estimating the effect of air pollution on road safety using atmospheric temperature inversions. *Journal of Environmental Economics and Management* **98**, 102250 (2019).
53. Running With a Mask? The Effect of Air Pollution on Marathon Runners' Performance - Mengmeng Guo, Shihe Fu, 2019. https://journals.sagepub.com/doi/abs/10.1177/1527002518822701.
54. Air Quality and Error Quantity: Pollution and Performance in a High-Skilled, Quality-Focused Occupation | Journal of the Association of Environmental and Resource Economists: Vol 5, No 4. https://www.journals.uchicago.edu/doi/full/10.1086/698728.
55. Heyes, A., Rivers, N. & Schaufele, B. Pollution and Politician Productivity: The Effect of PM on MPs. *Land Economics* **95**, 157–173 (2019).
56. Künn, S., Palacios, J. & Pestel, N. *Indoor Air Quality and Cognitive Performance*. *IZA Discussion Papers* https://ideas.repec.org/p/iza/izadps/dp12632.html (2019).
57. Zhang, X., Zhang, X. & Chen, X. Happiness in the Air: How Does a Dirty Sky Affect Mental Health and Subjective Well-being? *J Environ Econ Manage* **85**, 81–94 (2017).
58. Heyes, A. & Zhu, M. Air pollution as a cause of sleeplessness: Social media evidence from a panel of Chinese cities. *Journal of Environmental Economics and Management* **98**, (2019).
59. Bondy, M., Roth, S. & Sager, L. Crime Is in the Air: The Contemporaneous Relationship between Air Pollution and Crime. *Journal of the Association of Environmental and Resource Economists* **7**, 555–585 (2020).

60. Burkhardt, J. *et al.* The effect of pollution on crime: Evidence from data on particulate matter and ozone. *Journal of Environmental Economics and Management* **98**, (2019).
61. Herrnstadt, E., Heyes, A., Muehlegger, E. & Saberian, S. Air Pollution and Criminal Activity: Microgeographic Evidence from Chicago. *American Economic Journal: Applied Economics* **13**, 70–100 (2021).
62. Chang, T. Y., Graff Zivin, J., Gross, T. & Neidell, M. The Effect of Pollution on Worker Productivity: Evidence from Call Center Workers in China. *American Economic Journal: Applied Economics* **11**, 151–172 (2019).
63. Hanna, R. & Oliva, P. *The Effect of Pollution on Labor Supply: Evidence from a Natural Experiment in Mexico City*. https://www.nber.org/papers/w17302 (2011) doi:10.3386/w17302.
64. Dasgupta, S. *et al.* Effects of climate change on combined labour productivity and supply: an empirical, multi-model study. *The Lancet Planetary Health* **5**, e455–e465 (2021).
65. Ostro, B. D. Air pollution and morbidity revisited: A specification test. *Journal of Environmental Economics and Management* **14**, 87–98 (1987).
66. European Commission. Joint Research Centre. Institute for Prospective Technological Studies. *GEM-E3 model documentation.* (Publications Office, 2013).
67. Aguiar, A., Chepeliev, M., Corong, E. L., McDougall, R. & Mensbrugghe, D. van der. The GTAP Data Base: Version 10. *Journal of Global Economic Analysis* **4**, 1–27 (2019).
68. Vandyck, T. *et al.* Air quality co-benefits for human health and agriculture counterbalance costs to meet Paris Agreement pledges. *Nat Commun* **9**, 4939 (2018).
