# Supplementary material for: The global and regional air quality impacts of dietary change
Source: Nat Commun. 2023 Oct 6;14:6227. doi: 10.1038/s41467-023-41789-3 (PMC10558460; doi:10.1038/s41467-023-41789-3)
Supplement: Supplementary file 2 — Description of Additional Supplementary Files [file 41467_2023_41789_MOESM2_ESM.pdf]

Description of Additional Supplementary Files

File Name: Supplementary Data 1
Description: Global and regional estimates of impacts. The datafile contains the global and regional estimates of food consumption, production, agricultural emissions, air pollution, mortality impacts, and economic impacts associated with dietary changes, as displayed in the manuscript and supplementary information.

File Name: Supplementary Data 2
Description: Country-level estimates of impacts. The datafile contains country-level estimates of the air pollution, mortality, and economic impacts of dietary changes.
